# Propagation of elastic waves through textured polycrystals: application to ice

**DOI:** 10.1098/rspa.2014.0988

**Published:** 2015-05-08

**Authors:** Agnès Maurel, Fernando Lund, Maurine Montagnat

**Affiliations:** 1 Institut Langevin, CNRS, ESPCI ParisTech, Paris 75005, France; 2 Departamento de Física, Facultad de Ciencias Físicas y Matemáticas, Universidad de Chile, Casilla 487-3, Santiago, Chile; 3 LGGE, CNRS, Université de Grenoble Alpes, Grenoble 38041, France

**Keywords:** effective medium, elastic wave propagation, polycrystal, ice

## Abstract

The propagation of elastic waves in polycrystals is revisited, with an emphasis on configurations relevant to the study of ice. Randomly oriented hexagonal single crystals are considered with specific, non-uniform, probability distributions for their major axis. Three typical textures or fabrics (i.e. preferred grain orientations) are studied in detail: one cluster fabric and two girdle fabrics, as found in ice recovered from deep ice cores. After computing the averaged elasticity tensor for the considered textures, wave propagation is studied using a wave equation with elastic constants *c*=〈*c*〉+*δc* that are equal to an average plus deviations, presumed small, from that average. This allows for the use of the Voigt average in the wave equation, and velocities are obtained solving the appropriate Christoffel equation. The velocity for vertical propagation, as appropriate to interpret sonic logging measurements, is analysed in more details. Our formulae are shown to be accurate at the 0.5% level and they provide a rationale for previous empirical fits to wave propagation velocities with a quantitative agreement at the 0.07–0.7% level. We conclude that, within the formalism presented here, it is appropriate to use, with confidence, velocity measurements to characterize ice fabrics.

## 1. Introduction

The propagation of sound in polycrystals has long been the subject of studies (for a review, see [1]). Polycrystals are inhomogeneous media, the inhomogeneity being in the size and/or in the crystallographic orientations of the grains. To describe the propagation of elastic waves through a large number of grains, averaged wave properties have to be determined and, until the 1980s, these properties were determined by considering a spatial average. This average was obtained from the inverse of the velocity following the intuitive argument that the time of flight is the correct ‘intensive’ variable to consider: the time of flight needed to travel along a given distance is equal to the sum of the times needed to go through subsequent grains on the wave path [2,3]. However, this intuitive approach neglects the mode conversion between longitudinal and transverse waves at the grain boundaries, and it does not guarantee that the obtained velocities respect the symmetries of the polycrystal. Alternatively, velocities have been derived using averaged elasticity parameters in the static limit, that is, interrogating the average of the stress to strain relation, rather than the wave equation (the wave velocity is a sub-result of the primary average). Thus, the square of the velocity (Voigt average) or the square of the inverse of the velocity (Reuss average) have been considered, and it is commonly accepted that these two average values bound the observed or measured values. Most of these studies considered the case of randomly oriented grains, with a uniform distribution of orientations, that is, an isotropic fabric in which preferred orientations were disregarded, and they used an average for the longitudinal wave and for the transverse waves independently, thus again neglecting mode conversion [4]. The influence of microstructure on the macroscopic properties of random heterogeneous materials has been surveyed by Torquato [5].

Starting with Karal and Keller in the 1960s [6,7], stochastic methods have been developed in which the spatial averages are replaced by ensemble averages [8]. These statistical approaches simplify the problem in a mathematically rigorous formalism and it is widely accepted that the assumption of ergodicity in the stochastic process ensures that the two averages, spatial average and ensemble average, are equivalent [9]. The basic idea behind ergodicity is that, for a sufficiently long wave propagation distance, all the realizations of the averaged quantity (for instance, all possible crystallographic orientations) will be interrogated by the wave. Note that, if this is not the case, a spatial average is questionable as well. Indeed, in that case, the apparent velocity along a short propagation distance will be dependent on the particular realization, that is, dependent on the states (size, crystallographic orientations, etc.) of a small number of grains. Rather, spatial or ensemble averages have to refer to the most probable realization, to which any particular single realization is believed to be close. Nowadays, the statistical approach is the method used in most studies. An important advantage is that a perturbative method can be properly iterated to extract information on the effective medium, such as the attenuation and the backscattering, which appear as a second-order effect, that cannot be obtained by simple-minded averaging. The successive iterations are often referred to as the successive Born approximations [1,10,11] or the successive approximations of the Dyson equation [12–15].

Textured polycrystals are structures which have crystallized with a preferred orientation or which have been loaded by a non-uniform stress after formation. This latter case concerns notably ice polycrystals, as recovered in ice sheets along deep ice cores, which is the motivation of this study. We consider hexagonal single crystals, the predominant form of ice found on Earth. As usual, we denote by *c* the long axis of the unit cell and by *a* the short axis on the plane perpendicular to *c*. In the central part of deep ice sheets, ice is deformed at very low strain rates (10^−10^ to 10^−12^ s^−1^) induced by progressive accumulation of snow layers at the surface. In the simplest case (for instance, under perfect domes), ice is loaded by uniaxial compression due to gravity, and the fabric evolves from nearly isotropic at the surface towards a cluster fabric, where the *c*-axes are randomly distributed within a cone with vertical axis. The cone angle decreases with depth or ice age, until a strong single maximum close to the bedrock is reached [16,17]. At the scale of thousands of years, ice may also flow along flow lines that depend on a deformation field that is mainly influenced by the surface slope and the bedrock properties, and also because a geographical dome can shift in position [18]. Under these conditions, ice fabrics result from a complex stress field, the *c*-axes rotating towards the axes of compressive stress and away from the axes of tensional stress (or equivalently the *a*-axes in the basal plane tend to be aligned with the tensional axis). Among the many possible fabrics, girdle fabrics (defined below) are characteristic of ice encountered in convergent flow regions, where the vertical axis corresponds to the compression due to gravity and with a tensional axis due to a given ice flow direction [19]. This is the case for ice along a ridge, a few hundred metres from a geographical dome; here, the dissymmetry in the surface slopes along and perpendicular to the ridge can induce a horizontal tension component in the strain field [20].

Until recently, this fabric was mostly determined by recovering the individual grain orientations on an ice thin section (dimensions of about 10×10 cm^2^ or 100– 500 grains) and this method is certainly the most precise when a statistically sufficient number of grains can be found in the thin section. An alternative approach was initiated in the 1970s by Bennett [21] (see also [22], ch. 6) and Bentley [23]: the use of borehole sonic measurements to determine *in situ* depth-continuous fabric. This method has been tested at Dome C, East Antarctica [24], and it reveals the sensitivity of both the longitudinal and transverse waves with depth, and thus, possibly, the variation of ice fabrics with depth. However, uncertainties appear in the inversion process as used in [24], which are partially due to the measurements themselves but which are also due to the model of the sonic velocities that is employed (this latter point will be discussed in this study). Thus, there is a need to provide accurate models for these media to answer the question of whether or not sonic logging is able to discriminate the different fabrics and, within a given fabric, to determine the degree of anisotropy. This is the goal of this paper, where the propagation of elastic waves in polycrystals with cluster and girdle fabrics is studied. Note that the presence of stress may affect the wave speeds (acoustoelastic effect) but it is excluded from consideration in our study.

Section 2 presents the definition and derivation of the second-order orientation tensor, whose maximum eigenvalue is a common characteristic of the degree of anisotropy of ice polycrystals [17]. This is done in order to get closed forms of the orientation tensors by defining simple orientation distribution functions (ODFs) of the *c*-axes for cluster and girdle fabrics. The realism of these ODFs with respect to existing physical modelling of the fabrics (see [25,26]) will be discussed elsewhere. In §3, we derive the Voigt matrices for the considered fabrics, starting by averaging the tensors of a single crystal expressed in an arbitrary coordinate frame. The expected effective anisotropies are obtained: hexagonal with vertical transverse isotropy (VTI) for clusters, orthorhombic for partial girdles and hexagonal with horizontal transverse isotropy (HTI) for thick girdles. Direct application of these calculations consists in setting the determinant of the Christoffel matrix equal to zero and this is presented in §4 for an arbitrary direction of the wave propagation. Except in the case of the partial girdle, closed forms for the velocities for the P-wave and for the S-waves are obtained. Because our calculations are primary motivated by their application in glaciology, we collect in §5 elements of discussion in this particular context. This concerns notably the inspection of the accuracy of our approximation, which assumes that the local deviation *δc*_*ijkl*_ of the elastic stiffness is small with respect to its average value. In the Introduction and throughout the paper, we use interchangeably the term anisotropy for the anisotropy (in its purest form) of the single crystals and for the effective anisotropy characteristic of the fabric, both resulting from a homogenization process (at the scale of many atoms and at the scale of many grains). Finally, when used, the values of the elastic constants for ice single crystals are taken from [21] and reported below

1.1 $$\text{ice single crystal}\left\{\begin{array}{l} A=14.06\times 10^{9}\;{N\;m}^{-2},\;C=15.24\times 10^{9}\;{N\;m}^{-2},\\ L=3.06\times 10^{9}\;{N\;m}^{-2},\;N=3.455\times 10^{9}\;{N\;m}^{-2},\\ F=5.88\times 10^{9}\;{N\;m}^{-2},\;\rho =917\;\mathrm{kg}\;m^{-3}, \end{array}\right.$$

where (*A*,*C*,*L*,*N*,*F*) are the elastic parameters of the elasticity tensor (see equation (3.3)).

## 2. Orientation distribution function and second-order orientation tensor

It is usual to characterize the anisotropy of ice polycrystals using the second-order orientation tensor a defined by

**Figure 1.**
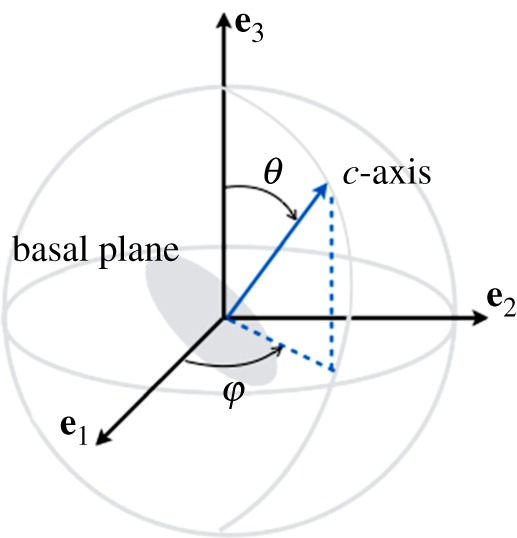
Orientation (*φ*,*θ*) of the *c*-axis in the frame (**e**_1_,**e**_2_,**e**_3_). The basal plane containing the *a*-axes is perpendicular to the *c*-axis.

**Figure 2.**
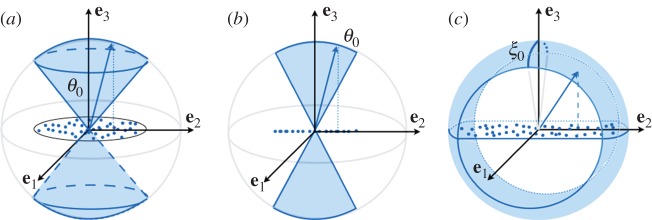
(*a*) Clustered ice fabrics resulting in VTI symmetry and (*b*,*c*) girdle fabrics. (*b*) Partial girdle resulting in orthorhombic symmetry and (*c*) thick girdle resulting in HTI symmetry. Dotted points in the (**e**_1_,**e**_2_) plane correspond to the projections of possible *c*-axes in the Schmidt net.

2.1 $$a_{ij}=\langle c_{i}c_{j}\rangle ,$$

where 〈.〉 denotes ensemble averages. That is, an average over all possible realizations of the directions of the *c*-axis [27]. In this section, we define the possible orientations of the *c*-axis using the ODF associated with the angles *θ* and *φ*, the co-latitude and longitudinal angles for cluster and girdle fabrics (figure 1); within girdle fabrics, we have restricted our study to the cases of partial and girdle fabrics (e.g. [28]). These three fabrics are relatively simple as they are defined by a single parameter in the ODF (the parameter is *θ*_0_ for the cluster fabric and for the partial girdle and it is *ξ*_0_ for the thick girdle; figure 2). Obviously, more complex fabrics may involve two or more parameters [26,29].

The largest eigenvalue of a, denoted *a*_1_ in the following, will be used further to characterize in a unified way the strength of the anisotropy of the resulting structure (limiting cases being *a*_1_=1/3 for a fully isotropic structure and *a*_1_=1 for a fabric with a single maximum; that is, when all the *c*-axes have the same direction) [27]. From figure 1, one can anticipate that cluster and thick girdle fabrics correspond to a resulting structure with hexagonal symmetry, whereas the partial girdle corresponds to a resulting structure with orthorhombic symmetry. In the case of the clusters, the structure is invariant by rotation along the vertical axis **e**_3_, thus it is isotropic in the transverse plane (often referred to as VTI for vertical transverse isotropy). In the case of a thick girdle, the structure is isotropic in a plane perpendicular to a horizontal axis (**e**_1_ in figure 2*c*, referred to as HTI for horizontal transverse isotropy).

### (a) Cluster fabric

Clustered fabrics, figure 1*a*, correspond to *c*-axes being oriented within a cone about the vertical axis **e**_3_ and we denote *θ*_0_ the opening angle this cone makes with the vertical. This configuration is naturally described by the spherical angles (*φ*,*θ*) (figure 1), with the *c*-axis defined by

2.2 $$\text{c}=\left(\begin{array}{c} \sin\theta \cos\varphi \\ \sin\theta \sin\varphi \\ \cos\theta \end{array}\right),$$

with *φ*∈[0,2*π*] and *θ*∈[0,*θ*_0_]. The probability distribution function *p*(*φ*,*θ*) is, as in [22,27],

2.3 $$p(\varphi ,\theta )=\frac{H_{1}(\theta )}{2\pi (1-\cos\theta_{0})},$$

with *H*_1_(*θ*)=1, for 0≤*θ*≤*θ*_0_ and zero otherwise. For the spherical angles, the average of any quantity *A* is defined by

2.4 $$\langle A\rangle =\int_{0}^{2\pi }d\varphi \int_{0}^{\pi /2}d\theta \sin\theta p(\varphi ,\theta )A.$$

It follows, using equations (2.1)–(2.4), that the second orientation tensor a is given as (denoting (*sθ*, *cθ*) for (sin⁡θ,cos⁡θ), respectively, (*cφ*,*sφ*) for (cos⁡φ,sin⁡φ))

2.5 $$a=\frac{1}{2\pi (1-\cos\theta_{0})}\int_{0}^{2\pi }d\varphi \int_{0}^{\theta_{0}}d\theta \left(\begin{array}{ccc} \text{s}\theta^{3}\text{c}\varphi^{2} & \text{s}\theta^{3}\text{c}\varphi \text{s}\varphi & \text{s}\theta^{2}\text{c}\theta \text{c}\varphi \\ \text{s}\theta^{3}\text{c}\varphi \text{s}\varphi & \text{s}\theta^{3}\text{s}\varphi^{2} & \text{s}\theta^{2}\text{c}\theta \text{s}\varphi \\ \text{s}\theta^{2}\text{c}\theta \text{c}\varphi & \text{s}\theta^{2}\text{c}\theta \text{s}\varphi & \text{c}\theta^{2}\text{s}\theta \end{array}\right)=\left(\begin{array}{ccc} a_{3} & 0 & 0\\ 0 & a_{2} & 0\\ 0 & 0 & a_{1} \end{array}\right),$$

with

2.6 $$\left.\begin{array}{rl} a_{1} & =\frac{1}{3}[1+\cos\theta_{0}+\cos^{2}\theta_{0}]\\ a_{2} & =a_{3}=\frac{1}{6}[2-\cos\theta_{0}-\cos^{2}\theta_{0}], \end{array}\right\}$$

in agreement with [22,27]. We have ordered the eigenvalues *a*_1_>*a*_2_=*a*_3_, and *a*_2_=*a*_3_ is obtained because the directions along **e**_1_ and **e**_2_ are equivalent for the VTI symmetry. The cluster fabrics cover all the degrees of anisotropy, from full isotropy with *a*_1_=1/3 (*θ*_0_=*π*/2, corresponding to all possible orientations of the *c*-axes) to the maximum anisotropy, with *a*_1_=1 (*θ*_0_=0, corresponding to all the *c*-axes being aligned with **e**_3_, often referred to as a single-maximum fabric).

### (b) Partial girdle

This configuration is two dimensional (figure 2*b*), and the polar angle *θ* is sufficient to describe the orientation of the *c*-axis

2.7 $$c=\left(\begin{array}{c} 0\\ \pm \sin\theta \\ \cos\theta \end{array}\right),$$

for *θ*∈[0,*θ*_0_] (the ± signs correspond to *φ*=*π*/2(+) and 3*π*/2(−)). The problem being two dimensional in (**e**_2_,**e**_3_), the projection onto (**e**_1_,**e**_2_) is not needed and the average is defined with *θ* being considered as a polar angle. For any quantity *A*, we now have

2.8 $$\langle A\rangle =\int_{0}^{2\pi }d\varphi \int_{0}^{\pi /2}d\theta p(\varphi ,\theta )A,$$

and we have kept *φ* in the average as the plane of rotation of the *c*-axes has to be defined by a delta-function to fix the *φ*-values, with

2.9 $$p(\varphi ,\theta )=\frac{1}{\theta_{0}}\left[\delta \left(\varphi -\frac{\pi }{2}\right)+\delta \left(\varphi -\frac{3\pi }{2}\right)\right]H_{2}(\theta )$$

and *H*_2_(*θ*)=1 for *θ* in [0,*θ*_0_], zero otherwise. Note that we could use *θ*∈[−*θ*_0_,*θ*_0_] and *φ*=*π*/2 but the calculations shown below are easier using the form above. From equations (2.7) and (2.8), and summing the two contributions of the *c*-axes for *φ*=*π*/2 and 3*π*/2 (with projections ±sin⁡θ on **e**_2_), a is diagonal, with

2.10 $$a=\frac{1}{\theta_{0}}\int_{0}^{\theta_{0}}d\theta \left(\begin{array}{ccc} 0 & 0 & 0\\ 0 & \text{s}\theta^{2} & 0\\ 0 & 0 & \text{c}\theta^{2} \end{array}\right)=\left(\begin{array}{ccc} a_{3} & 0 & 0\\ 0 & a_{2} & 0\\ 0 & 0 & a_{1} \end{array}\right)$$

and

2.11 $$a_{1}=\frac{1}{2}[1+\text{sinc}2\theta_{0}],\;a_{2}=\frac{1}{2}[1-\text{sinc}2\theta_{0}],\;a_{3}=0,$$

where we have defined sincx≡sin⁡x/x, and sinc0=1. As we could have anticipated, the partial girdle is always anisotropic, with *a*_1_ varying between $a_{1}=\frac{1}{2}$ (perfect girdle, for *θ*_0_=*π*/2) and *a*_1_=1 (single maximum fabric for *θ*_0_=0).

#### (i) Thick girdle

**Figure 3.**
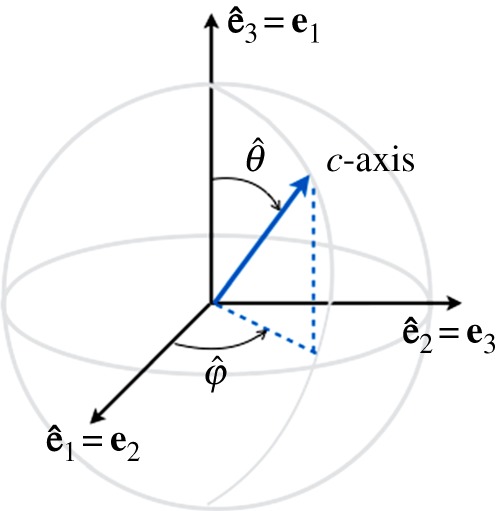
Parametrization angles $\left(\hat{\varphi },\hat{\theta }\right)$ used for the thick girdle and the correspondences between the coordinate frames $\left({\hat{\text{e}}}_{1},{\hat{\text{e}}}_{2},{\hat{\text{e}}}_{3}\right)$ and (**e**_1_,**e**_2_,**e**_3_).

The thick girdle is parametrized by two angles but, as defined, *θ* and *φ* are not adapted to deal with it in a simple way: indeed, as defined, *θ*∈[*π*/2−*ξ*_0_;*π*/2+*ξ*_0_] and *φ*∈[0;2*π*] describe a thick girdle with VTI symmetry (symmetry by rotation with respect to **e**_3_); the thick girdle has HTI symmetry, with a symmetry by rotation with respect to **e**_1_. To avoid additional calculations in the forthcoming calculations of the Voigt matrix (see §3), we use the following ‘trick’. (i) We denote ($\hat{\theta },\hat{\varphi })$ the co-latitude and longitudinal angles in a frame $\left({\hat{\text{e}}}_{1},{\hat{\text{e}}}_{2},{\hat{\text{e}}}_{3}\right)$, as previously (figure 3); (ii) the ODF is defined to produce a thick girdle with rotational invariance around ${\hat{\text{e}}}_{3}$, thus $\hat{\theta }\in [\pi /2-\xi_{0};\pi /2+\xi_{0}]$ and $\hat{\varphi }\in [0;2\pi ]$; and (iii) the characteristics of the thick girdle with rotational invariance around **e**_1_ are simply deduced by defining

2.12 $${\text{e}}_{1}={\hat{\text{e}}}_{3},\;{\text{e}}_{2}={\hat{\text{e}}}_{1}\;\mathrm{and}\;{\text{e}}_{3}={\hat{\text{e}}}_{2}.$$

Now, the *c*-axis is parametrized by $\hat{\theta }$ and $\hat{\varphi }$ in equation (2.2) when expressed in terms of $\left({\hat{\text{e}}}_{1},{\hat{\text{e}}}_{2},{\hat{\text{e}}}_{3}\right)$. When expressed in terms of (**e**_1_,**e**_2_,**e**_3_), it is

2.13 $$c=\left(\begin{array}{c} \cos\hat{\theta }\\ \sin\hat{\theta }\cos\hat{\varphi }\\ \sin\hat{\theta }\sin\hat{\varphi } \end{array}\right),$$

with $\hat{\varphi }\in [0,2\pi ]$ and $\hat{\theta }\in [\pi /2-\xi_{0},\pi /2+\xi_{0}]$. Averages are defined by

2.14 $$\langle A\rangle =\int_{0}^{2\pi }d\hat{\varphi }\int_{0}^{\pi }d\hat{\theta }\sin\hat{\theta }p(\hat{\theta },\hat{\xi })A,$$

with the simple ODF

2.15 $$p(\hat{\varphi },\hat{\theta })=\frac{1}{4\pi \sin\xi_{0}}H_{3}(\hat{\theta }),$$

and $H_{3}(\hat{\theta })=1$ for $\hat{\theta }\in [\pi /2-\xi_{0},\pi /2+\xi_{0}]$, and zero otherwise. The *c*-axis is given by equations (2.13), and, using equations (2.14) and (2.15), the a tensor is again diagonal

2.16 $$a=\frac{1}{4\pi \sin\xi_{0}}\int_{0}^{2\pi }d\hat{\varphi }\int_{\pi /2-\xi_{0}}^{\pi /2+\xi_{0}}d\hat{\theta }\left(\begin{array}{ccc} \text{s}\hat{\theta }\text{c}{\hat{\theta }}^{2} & \text{c}\hat{\theta }\text{s}{\hat{\theta }}^{2}\text{c}\hat{\varphi } & \text{c}\hat{\theta }\text{s}{\hat{\theta }}^{2}\text{s}\hat{\varphi }\\ \text{c}\hat{\theta }\text{s}{\hat{\theta }}^{2}\text{c}\hat{\varphi } & \text{s}{\hat{\theta }}^{3}\text{c}{\hat{\varphi }}^{2} & \text{s}{\hat{\theta }}^{2}\text{s}{\hat{\varphi }}^{2}\text{c}\hat{\varphi }\\ \text{c}\hat{\theta }\text{s}\hat{\theta }\text{s}{\hat{\varphi }}^{2} & \text{s}{\hat{\theta }}^{3}\text{s}\hat{\varphi }\text{c}\hat{\varphi } & \text{s}{\hat{\theta }}^{2}\text{s}{\hat{\varphi }}^{3} \end{array}\right)=\left(\begin{array}{ccc} a_{3} & 0 & 0\\ 0 & a_{1} & 0\\ 0 & 0 & a_{1} \end{array}\right),$$

with

2.17 $$a_{1}=a_{2}=\frac{1}{2}\left[1-\frac{\sin^{2}\xi_{0}}{3}\right],\;a_{3}=\frac{1}{3}\sin^{2}\xi_{0}.$$

As for the clustered fabrics, two of the eigenvalues are equal. This reflects the equivalence between two of the principal directions of the structure (**e**_2_ and **e**_3_ for the thick girdle). As expected, the thick girdles prolongate the range of anisotropy of the partial girdles, with *a*_1_ varying from *a*_1_=1/2 (perfect partial girdle for *ξ*_0_=0) to *a*_1_=1/3 (perfect isotropy for *ξ*_0_=*π*/2).

### (c) Evolution of the anisotropy with the *a*_*i*_

**Figure 4.**
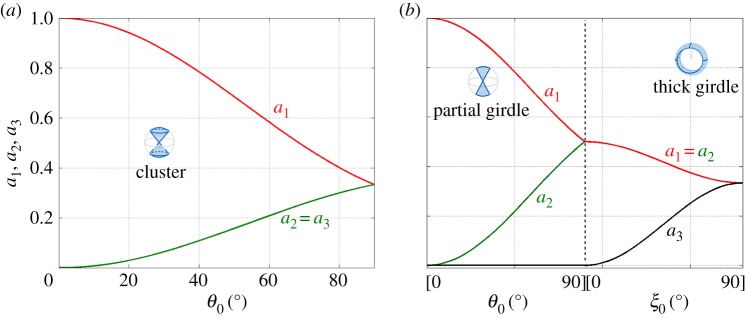
Eigenvalues (*a*_1_,*a*_2_,*a*_3_) of the second-order orientation tensor a. (*a*) As a function of *θ*_0_, for the cluster (equation (2.6)), and (*b*) as a function of *θ*_0_ for the girdle (equation (2.11)), and as a function of *ξ*_0_ for the thick girdle (equation (2.17)).

Figure 4 shows the variation of the *a*_*i*_, *i*=1,2,3, as a function of *θ*_0_ for the cluster fabric and as a function of *θ*_0_ and *ξ*_0_ for the partial and thick girdles, respectively. Inspecting the value of *a*_1_ allows us to characterize the degree of anisotropy of a polycrystalline structure in a unified way, that is, independently of the considered fabric.

## 3. Elasticity tensors of single crystals and textured polycrystals

In this section, we derive the elasticity tensor *c*_*ijkl*_ of a single crystal when expressed in an arbitrary coordinate frame, with the *c*-axis being parametrized by the two spherical angles (*θ*,*φ*). The result is further used to derive the elasticity tensors for the cluster and girdle fabrics, describing average macroscopic properties of the polycrystalline materials. We use the elasticity tensor in Voigt's notation [30], *C*_*IJ*_ with the standard correspondences *c*_*ijk*ℓ_→*C*_*IJ*_, for (*i*,*j*)→*I*, (*k*,*l*)→*J* and (1,1)→1, (2,2)→2, (3,3)→3, (3,2),(2,3)→4, (3,1),(1,3)→5 and (1,2),(2,1)→6.

### (a) The elasticity tensor of a single crystal

The elasticity tensor is derived for an arbitrary direction of the *c*-axis (equation (2.2)) in (**e**_1_,**e**_2_,**e**_3_) (figure 1). The crystal frame $\left({\text{e}}_{1}^{0},{\text{e}}_{2}^{0},{\text{e}}_{3}^{0}\right)$ is defined by the principal directions of anisotropy, ${\text{e}}_{1}^{0}$, ${\text{e}}_{2}^{0}$ being in the basal plane and ${\text{e}}_{3}^{0}\equiv c$. In the reference frame (**e**_1_,**e**_2_,**e**_3_), we have

3.1 $${\text{e}}_{1}^{0}=\left(\begin{array}{c} \cos\theta \cos\varphi \\ \cos\theta \sin\varphi \\ -\sin\theta \end{array}\right),\;{\text{e}}_{2}^{0}=\left(\begin{array}{c} -\sin\varphi \\ \cos\varphi \\ 0 \end{array}\right)\;\mathrm{and}\;\text{c}={\text{e}}_{3}^{0}=\left(\begin{array}{c} \sin\theta \cos\varphi \\ \sin\theta \sin\varphi \\ \cos\theta \end{array}\right),$$

and we define the rotation matrix R

3.2 $$R\equiv \left(\begin{array}{ccc} \cos\theta \cos\varphi & \cos\theta \sin\varphi & -\sin\theta \\ -\sin\varphi & \cos\varphi & 0\\ \sin\theta \cos\varphi & \sin\theta \sin\varphi & \cos\theta \end{array}\right).$$

Note a non-conventional form of the rotation matrix when compared with the rotation matrix that takes Cartesian to spherical coordinates; indeed, in this usual notation, the vector **e**_1_ is transformed into the radial vector, which is the first vector in spherical coordinates, but for convenience it is the third vector ${\text{e}}_{3}^{0}$ in the present case. This produces a *c*-axis oriented along the vertical direction **e**_3_ for *φ*=*θ*=0.

When expressed in the local frame $\left({\text{e}}_{1}^{0},{\text{e}}_{2}^{0},{\text{e}}_{3}^{0}\right)$, the single crystal with hexagonal symmetry is described by the elasticity tensor $c_{ijkl}^{0}$, written as C^0^ in Voigt's notation,

3.3 $$C^{0}=\left(\begin{array}{cccccc} A & A-2N & F & 0 & 0 & 0\\ A-2N & A & F & 0 & 0 & 0\\ F & F & C & 0 & 0 & 0\\ 0 & 0 & 0 & L & 0 & 0\\ 0 & 0 & 0 & 0 & L & 0\\ 0 & 0 & 0 & 0 & 0 & N \end{array}\right).$$

In a frame (**e**_1_,**e**_2_,**e**_3_), the elasticity tensor *c*_*abcd*_ is deduced from $c_{ijkl}^{0}$ using

3.4 $$c_{abcd}=R_{ia}R_{jb}R_{kc}R_{ld}c_{ijk\ell }^{0},$$

according to the transformation laws for tensors (see, for example, [31] for the calculation of anisotropic properties from texture data using an open source package). The corresponding relation between the Voigt matrices C^0^ and C is in general involved, and the use of the Bond matrix is often preferred [32]. We do not use the Bond matrix formalism but, owing to the hexagonal symmetry in C^0^, we use a reasonably tractable expression, similar to the one used in [33],

3.5 $$\begin{array}{rl} c_{abcd} & =A[R_{1a}R_{1b}+R_{2a}R_{2b}][R_{1c}R_{1d}+R_{2c}R_{2d}]+CR_{3a}R_{3b}R_{3c}R_{3d}\\ & \;+N[(R_{1a}R_{2b}+R_{2a}R_{1b})(R_{1c}R_{2d}+R_{2c}R_{1d})-2(R_{1a}R_{1b}R_{2c}R_{2d}+R_{2a}R_{2b}R_{1c}R_{1d})]\\ & \;+L[(R_{1a}R_{3b}+R_{3a}R_{1b})(R_{1c}R_{3d}+R_{3c}R_{1d})+(R_{2a}R_{3b}+R_{3a}R_{2b})(R_{2c}R_{3b}+R_{3c}R_{2b})]\\ & \;+F[(R_{1a}R_{1b}+R_{2a}R_{2b})R_{3c}R_{3d}+R_{3a}R_{3b}(R_{1c}R_{1d}+R_{2c}R_{2d})]. \end{array}$$

These terms are calculated and we obtain, for the diagonal terms,

3.6 $$\left.\begin{array}{rl} C_{11} & =A{\left[1-\text{s}\theta^{2}\text{c}\varphi^{2}\right]}^{2}+C\text{c}\varphi^{4}\text{s}\theta^{4}+2(2L+F)\text{s}\theta^{2}\text{c}\varphi^{2}[1-\text{s}\theta^{2}\text{c}\varphi^{2}],\\ C_{22} & =A{\left[1-\text{s}\theta^{2}\text{s}\varphi^{2}\right]}^{2}+C\text{s}\varphi^{4}\text{s}\theta^{4}+2(2L+F)\text{s}\theta^{2}\text{s}\varphi^{2}[1-\text{s}\theta^{2}\text{s}\varphi^{2}],\\ C_{33} & =A\text{s}\theta^{4}+2(2L+F)\text{s}\theta^{2}\text{c}\theta^{2}+C\text{c}\theta^{4},\\ C_{44} & =(A+C-2F)\text{s}\theta^{2}\text{c}\theta^{2}\text{s}\varphi^{2}+L[\text{s}\varphi^{2}{\left(\text{c}\theta^{2}-\text{s}\theta^{2}\right)}^{2}+\text{c}\theta^{2}\text{c}\varphi^{2}]+N\text{s}\theta^{2}\text{c}\varphi^{2},\\ C_{55} & =(A+C-2F)\text{s}\theta^{2}\text{c}\theta^{2}\text{c}\varphi^{2}+L[\text{c}\varphi^{2}{\left(\text{c}\theta^{2}-\text{s}\theta^{2}\right)}^{2}+\text{c}\theta^{2}\text{s}\varphi^{2}]+N\text{s}\theta^{2}\text{s}\varphi^{2},\\ C_{66} & =(A+C-2F)\text{s}\theta^{4}\text{c}\varphi^{2}\text{s}\varphi^{2}+L\text{s}\theta^{2}(1-4\text{s}\theta^{2}\text{s}\varphi^{2}\text{c}\varphi^{2})+N\text{c}\theta^{2}, \end{array}\right\}$$

and the off-diagonal terms

3.7 $$\left.\begin{array}{rl} C_{12} & =A(\text{c}\theta^{2}+\text{s}\theta^{4}\text{c}\varphi^{2}\text{s}\varphi^{2})+(C-4L)\text{s}\theta^{4}\text{s}\varphi^{2}\text{c}\varphi^{2}-2N\text{c}\theta^{2}+F\text{s}\theta^{2}(1-2\text{s}\theta^{2}\text{s}\varphi^{2}\text{c}\varphi^{2}),\\ C_{13} & =A\text{s}\theta^{2}(1-\text{s}\theta^{2}\text{c}\varphi^{2})+(C-4L)\text{s}\theta^{2}\text{c}\theta^{2}\text{c}\varphi^{2}-2N\text{s}\theta^{2}\text{s}\varphi^{2}+F[\text{c}\varphi^{2}(\text{s}\theta^{4}+\text{c}\theta^{4})+\text{s}\varphi^{2}\text{c}\theta^{2}],\\ C_{14} & =\text{s}\theta \text{c}\theta \text{s}\varphi [-A(1-\text{c}\varphi^{2}\text{s}\theta^{2})+(C-4L)\text{s}\theta^{2}\text{c}\varphi^{2}+2N+F(1-2\text{c}\varphi^{2}\text{s}\theta^{2})],\\ C_{15} & =\text{s}\theta \text{c}\theta \text{c}\varphi [-A(1-\text{c}\varphi^{2}\text{s}\theta^{2})+C\text{s}\theta^{2}\text{c}\varphi^{2}+(2L+F)(1-2\text{c}\varphi^{2}\text{s}\theta^{2})],\\ C_{16} & =\text{s}\theta^{2}\text{s}\varphi \text{c}\varphi [-A(1-\text{c}\varphi^{2}\text{s}\theta^{2})+C\text{s}\theta^{2}\text{c}\varphi^{2}+(2L+F)(1-2\text{c}\varphi^{2}\text{s}\theta^{2})],\\ C_{23} & =\text{s}\theta^{2}[A(1-\text{s}\varphi^{2}\text{s}\theta^{2})+(C-4L)\text{c}\theta^{2}\text{s}\varphi^{2}-2N\text{c}\varphi^{2}]+F[\text{c}\theta^{2}+\text{s}\varphi^{2}\text{s}\theta^{2}(2\text{s}\theta^{2}-1)],\\ C_{24} & =\text{c}\theta \text{s}\theta \text{s}\varphi [-A(1-\text{s}\theta^{2}\text{s}\varphi^{2})+C\text{s}\theta^{2}\text{s}\varphi^{2}+(2L+F)(1-2\text{s}\varphi^{2}\text{s}\theta^{2})],\\ C_{25} & =\text{c}\theta \text{s}\theta \text{c}\varphi [-A(1-\text{s}\theta^{2}\text{s}\varphi^{2})+(C-4L)\text{s}\theta^{2}\text{s}\varphi^{2}+2N+F(1-2\text{s}\theta^{2}\text{s}\varphi^{2})],\\ C_{26} & =\text{s}\varphi \text{c}\varphi \text{s}\theta^{2}[-A(1-\text{s}\varphi^{2}\text{s}\theta^{2})+C\text{s}\theta^{2}\text{s}\varphi^{2}+(2L+F)(1-2\text{s}\varphi^{2}\text{s}\theta^{2})],\\ C_{34} & =-\text{s}\varphi \text{s}\theta \text{c}\theta [A\text{s}\theta^{2}-C\text{c}\theta^{2}+(2L+F)(1-2\text{s}\theta^{2})],\\ C_{35} & =-\text{c}\varphi \text{s}\theta \text{c}\theta [A\text{s}\theta^{2}-C\text{c}\theta^{2}+(2L+F)(1-2\text{s}\theta^{2})],\\ C_{36} & =\text{s}\varphi \text{c}\varphi \text{s}\theta^{2}[-A\text{s}\theta^{2}+(C-4L)\text{c}\theta^{2}+2N+F(1-2\text{c}\theta^{2})],\\ C_{45} & =\text{s}\varphi \text{c}\varphi \text{s}\theta^{2}[(A+C-2F)\text{c}\theta^{2}+L(1-4\text{c}\theta^{2})-N],\\ C_{46} & =\text{c}\theta \text{s}\theta \text{c}\varphi [(A+C-2F)\text{s}\theta^{2}\text{s}\varphi^{2}+L(1-4\text{s}\theta^{2}\text{s}\varphi^{2})-N],\\ C_{56} & =\text{c}\theta \text{s}\theta \text{s}\varphi [(A+C-2F)\text{s}\theta^{2}\text{c}\varphi^{2}+L(1-4\text{s}\theta^{2}\text{c}\varphi^{2})-N]. \end{array}\right\}$$

### (b) Elasticity tensors of textured polycrystals: cluster and girdle fabrics

Next, we derive the averaged elasticity tensors that are needed for the description of the effective macroscopic properties of polycrystals with cluster or girdle fabrics. This is done by averaging the Voigt matrices of single crystals (equations (3.6) and (3.7)) using the ODF in equations (2.3), (2.9) and (2.15).

#### (i) The clustered fabric

The averages of the Voigt matrix with elements *C*_*ij*_ in equations (3.6) and (3.7) are averaged according to

3.8 $$\langle C_{ij}\rangle =\int_{0}^{2\pi }d\varphi \int_{0}^{\pi /2}d\theta \sin\theta p(\varphi ,\theta )C_{ij}$$

with the ODF for the clustered fabric in equation (2.3). It is shown in appendix A that the resulting Voigt matrix is associated with a structure with VTI, as expected

3.9 $$\left(\begin{array}{cccccc} \left\langle C_{11}\right\rangle & \left\langle C_{11}\rangle -2\langle C_{66}\right\rangle & \left\langle C_{13}\right\rangle & 0 & 0 & 0\\ \left\langle C_{11}\rangle -2\langle C_{66}\right\rangle & \left\langle C_{11}\right\rangle & \left\langle C_{13}\right\rangle & 0 & 0 & 0\\ \left\langle C_{13}\right\rangle & \left\langle C_{13}\right\rangle & \left\langle C_{33}\right\rangle & 0 & 0 & 0\\ 0 & 0 & 0 & \left\langle C_{44}\right\rangle & 0 & 0\\ 0 & 0 & 0 & 0 & \left\langle C_{44}\right\rangle & 0\\ 0 & 0 & 0 & 0 & 0 & \left\langle C_{66}\right\rangle \end{array}\right),$$

with the elastic constants

3.10 $$\mathrm{cluster}\left\{\begin{array}{rl} \left\langle C_{11}\right\rangle & =\frac{1}{120}[A(45+19X+9Y)+3C(15-7X+3Y)+2(2L+F)(15+X-9Y)],\\ \left\langle C_{33}\right\rangle & =\frac{1}{15}[A(15-7X+3Y)+3C(X+Y)+2(2L+F)(2X-3Y)],\\ \left\langle C_{44}\right\rangle & =\frac{1}{30}[(A+C-2F)(2X-3Y)+3L(5-X+4Y)+5N(3-X)],\\ \left\langle C_{66}\right\rangle & =\frac{1}{120}[(A+C-2F)(15-7X+3Y)+12L(5-X-Y)+40NX],\\ \left\langle C_{13}\right\rangle & =\frac{1}{30}[3A(5-X-Y)+(C-4L)(2X-3Y)-10N(3-X)+F(15+X+6Y)] \end{array}\right.$$

with

3.11 $$\left.\begin{array}{rl} X & \equiv 1+\cos\theta_{0}+\cos^{2}\theta_{0},\\ Y & \equiv \cos^{3}\theta_{0}+\cos^{4}\theta_{0}. \end{array}\right\}$$

(1) The single maximum fabrics for *θ*_0_=0 (*X*=3, *Y* =2) lead to 〈*C*_11_〉=*A*, 〈*C*_33_〉=*C*, 〈*C*_44_〉=*L*, 〈*C*_66_〉=*N* and 〈*C*_13_〉=*F*; the effective medium is, as expected, the same as a homogeneous medium with a unique orientation of the *c*-axis along **e**_3_, in agreement with the expression of the Voigt matrix C^0^ in equation (3.3).
(2) For *θ*_0_=*π*/2 (*X*=1, *Y* =0), the average is done for *c*-axes varying randomly over all directions, resulting in an effective isotropy; we obtain
  3.12 $$\mathrm{effectiveisotropy}\left\{\begin{array}{l} {\left\langle C_{11}\right\rangle }^{\mathrm{iso}}={\left\langle C_{33}\right\rangle }^{\mathrm{iso}}=\frac{1}{15}[8A+3C+4(2L+F)],\\ {\left\langle C_{44}\right\rangle }^{\mathrm{iso}}={\left\langle C_{66}\right\rangle }^{\mathrm{iso}}=\frac{1}{15}[A+C-2F+6L+5N], \end{array}\right.$$
  and 〈*C*_13_〉^iso^=〈*C*_11_〉^iso^−2〈*C*_44_〉^iso^, resulting in effective Lamé coefficients
  3.13 $$\left.\begin{array}{rl} \lambda_{\mathrm{eff}} & =\frac{1}{15}[6A+C-4L+8F-10N],\\ \mu_{\mathrm{eff}} & =\frac{1}{15}[A+C+6L-2F+5N]. \end{array}\right\}$$
  These elastic constants in the isotropic case agree with previous derivations, referred to as the Markham derivation [2] (see also [33], eqn (4.b)).

#### (ii) Partial girdle fabrics

The average of the Voigt matrix is carried out according to the two-dimensional configuration

3.14 $$\langle C_{ij}\rangle =\int_{0}^{2\pi }d\varphi \int_{0}^{\pi /2}d\theta p(\varphi ,\theta )C_{ij},$$

with *p* being defined in equation (2.9). Calculations are straightforward (see details in appendix B) and the resulting Voigt matrix is associated with a structure with orthorhombic symmetry

3.15 $$\left(\begin{array}{cccccc} \left\langle C_{11}\right\rangle & \left\langle C_{12}\right\rangle & \left\langle C_{13}\right\rangle & 0 & 0 & 0\\ \left\langle C_{12}\right\rangle & \left\langle C_{22}\right\rangle & \left\langle C_{23}\right\rangle & 0 & 0 & 0\\ \left\langle C_{13}\right\rangle & \left\langle C_{23}\right\rangle & \left\langle C_{33}\right\rangle & 0 & 0 & 0\\ 0 & 0 & 0 & \left\langle C_{44}\right\rangle & 0 & 0\\ 0 & 0 & 0 & 0 & \left\langle C_{55}\right\rangle & 0\\ 0 & 0 & 0 & 0 & 0 & \left\langle C_{66}\right\rangle \end{array}\right),$$

with

3.16 $$\mathrm{partialgirdle}\left\{\begin{array}{l} \langle C_{11}\rangle =A,\\ \langle C_{22}\rangle =\frac{1}{8}[A(3+4{\text{S}}_{2}+{\text{S}}_{4})+C(3-4{\text{S}}_{2}+{\text{S}}_{4})+2(2L+F)(1-{\text{S}}_{4})],\\ \langle C_{33}\rangle =\frac{1}{8}[A(3-4{\text{S}}_{2}+{\text{S}}_{4})+C(3+4{\text{S}}_{2}+{\text{S}}_{4})+2(2L+F)(1-{\text{S}}_{4})],\\ \langle C_{44}\rangle =\frac{1}{8}[(A+C-2F)(1-{\text{S}}_{4})+4L(1+{\text{S}}_{4})],\\ \langle C_{55}\rangle =\frac{1}{2}[L(1+{\text{S}}_{2})+N(1-{\text{S}}_{2})],\\ \langle C_{66}\rangle =\frac{1}{2}[L(1-{\text{S}}_{2})+N(1+{\text{S}}_{2})],\\ \langle C_{12}\rangle =\frac{1}{2}[(A-2N)(1+{\text{S}}_{2})+F(1-{\text{S}}_{2})],\\ \langle C_{13}\rangle =\frac{1}{2}[(A-2N)(1-{\text{S}}_{2})+F(1+{\text{S}}_{2})],\\ \langle C_{23}\rangle =\frac{1}{8}[(A+C-4L)(1-{\text{S}}_{4})+2F(3+{\text{S}}_{4})], \end{array}\right.$$

where we have defined

3.17 $${\text{S}}_{2}\equiv \text{sinc}2\theta_{0}\;\text{and}\;{\text{S}}_{4}\equiv \text{sinc}4\theta_{0},$$

and sinc x≡sin⁡x/x (and sinc 0=1). As previously noted, partial girdles always have isotropic structure. For *θ*_0_=0 (S_2_=S_4_=1), we recover the hexagonal symmetry of a single crystal with the *c*-axis along **e**_3_ (equation (3.3)). Also, for *θ*_0_=*π*/2 (S_2_=S_4_=0), the effective medium has hexagonal symmetry with HTI corresponding to a perfect girdle, with 〈*C*_23_〉^PG^=〈*C*_22_〉^PG^−2〈*C*_44_〉^PG^ and

3.18 $$\mathrm{perfectgirdle}\left\{\begin{array}{l} {\left\langle C_{11}\right\rangle }^{\mathrm{PG}}=A,\\ {\left\langle C_{22}\right\rangle }^{\mathrm{PG}}={\left\langle C_{33}\right\rangle }^{\mathrm{PG}}=\frac{1}{8}[3(A+C)+2(2L+F)],\\ {\left\langle C_{44}\right\rangle }^{\mathrm{PG}}=\frac{1}{8}[A+C-2F+4L],\\ {\left\langle C_{55}\right\rangle }^{\mathrm{PG}}={\left\langle C_{66}\right\rangle }^{\mathrm{PG}}=\frac{1}{2}[L+N],\\ {\left\langle C_{12}\right\rangle }^{\mathrm{PG}}={\left\langle C_{13}\right\rangle }^{\mathrm{PG}}=\frac{1}{2}[A-2N+F]. \end{array}\right.$$

#### (iii) The thick girdle

The elasticity tensor for the thick girdle is derived using the same trick that was previously used for the derivation of the second-order orientation tensor in §2*b*(i). It allows for a straightforward use of the expression of the elasticity tensor derived in §3*a* for single crystals. Namely,

(1) We denote ${\hat{C}}_{ab}$ the elements of the Voigt matrix in equations (3.6) and (3.7), expressed in the frame $\left({\hat{\text{e}}}_{1},{\hat{\text{e}}}_{2},{\hat{\text{e}}}_{3}\right)$, with the usual conventions of the co-latitude and longitudinal angles $\left(\hat{\theta },\hat{\varphi }\right)$ defined in figure 3.
(2) The averages are performed as for the a tensor (equations (2.14) and (2.15)) to obtain
  3.19 $$\langle {\hat{C}}_{ab}\rangle =\int_{0}^{2\pi }d\hat{\varphi }\int_{0}^{\pi }d\hat{\theta }\sin\hat{\theta }p(\hat{\theta },\hat{\xi }){\hat{C}}_{ab}.$$
(3) The Voigt matrix C_*ij*_ expressed in the material frame (**e**_1_,**e**_2_,**e**_3_) is rearranged according to correspondences ${\text{e}}_{1}={\hat{\text{e}}}_{3}$, ${\text{e}}_{2}={\hat{\text{e}}}_{1}$ and ${\text{e}}_{3}={\hat{\text{e}}}_{2}$, leading to
  3.20 $$\langle C_{ij}\rangle =\langle {\hat{C}}_{ab}\rangle ,\;\mathrm{with}\;i=1,2,3,4,5,6\;\mathrm{correspondingto}\;a=3,1,2,6,4,5\;(\mathrm{resp}.\;j,b).$$

It is shown in appendix C that the resulting Voigt matrix is associated with a structure with HTI symmetry (with respect to **e**_1_),

3.21 $$\left(\begin{array}{cccccc} \left\langle C_{11}\right\rangle & \left\langle C_{12}\right\rangle & \left\langle C_{12}\right\rangle & 0 & 0 & 0\\ \left\langle C_{12}\right\rangle & \left\langle C_{22}\right\rangle & \left\langle C_{22}\rangle -2\langle C_{44}\right\rangle & 0 & 0 & 0\\ \left\langle C_{12}\right\rangle & \left\langle C_{22}\rangle -2\langle C_{44}\right\rangle & \left\langle C_{22}\right\rangle & 0 & 0 & 0\\ 0 & 0 & 0 & \left\langle C_{44}\right\rangle & 0 & 0\\ 0 & 0 & 0 & 0 & \left\langle C_{55}\right\rangle & 0\\ 0 & 0 & 0 & 0 & 0 & \left\langle C_{55}\right\rangle \end{array}\right),$$

with

3.22 $$\left.\begin{array}{rl} \left\langle C_{11}\right\rangle & =\frac{1}{15}[A(15-10{\text{s}\xi_{0}}^{2}+3{\text{s}\xi_{0}}^{4})+3C{\text{s}\xi_{0}}^{4}+2(2L+F)(5{\text{s}\xi_{0}}^{2}-3{\text{s}\xi_{0}}^{4})],\\ \left\langle C_{33}\right\rangle & =\frac{1}{120}[A(45+10{\text{s}\xi_{0}}^{2}+9{\text{s}\xi_{0}}^{4})+3C(15-10{\text{s}\xi_{0}}^{2}+3{\text{s}\xi_{0}}^{4})+2(2L+F)(15+10{\text{s}\xi_{0}}^{2}-9{\text{s}\xi_{0}}^{4})],\\ \left\langle C_{44}\right\rangle & =\frac{1}{120}[(A+C-2F)(15-10{\text{s}\xi_{0}}^{2}+3{\text{s}\xi_{0}}^{4})+12L(5-{\text{s}\xi_{0}}^{4})+40N{\text{s}\xi_{0}}^{2}],\\ \left\langle C_{55}\right\rangle & =\frac{1}{30}[(A+C-2F)(5{\text{s}\xi_{0}}^{2}-3{\text{s}\xi_{0}}^{4})+3L(5-5{\text{s}\xi_{0}}^{2}+4{\text{s}\xi_{0}}^{4})+5N(3-{\text{s}\xi_{0}}^{2})],\\ \left\langle C_{12}\right\rangle & =\frac{1}{30}[3A(5-{\text{s}\xi_{0}}^{4})+(C-4L)(5{\text{s}\xi_{0}}^{2}-3{\text{s}\xi_{0}}^{4})-10N(3-{\text{s}\xi_{0}}^{2})+F(15-5{\text{s}\xi_{0}}^{2}+6{\text{s}\xi_{0}}^{4})], \end{array}\right\}$$

where

3.23 $$\text{s}\xi_{0}\equiv \sin\xi_{0}.$$

(4) Thick girdles (with *a*_1_ between 1/3 and 1/2) have in general a lower degree of anisotropy than partial girdles (*a*_1_ between 1/2 and 1). Nevertheless, the two structures coincide when realizing a perfect girdle (partial with *θ*_0_=*π*/2 and thick with *ξ*_0_=0); in that case, the effective parameters in equation (3.22) (with s*ξ*_0_=0) coincide with those in equation (3.18).
(5) The thick girdle becomes isotropic *ξ*_0_=*π*/2 (s*ξ*_0_=1), and we indeed recover the effective isotropic parameters (equation (3.12)).

## 4. Wave propagation in a textured polycrystal

In this section, we derive the velocities of elastic waves propagating in polycrystals (with cluster or girdle fabrics). Using the Voigt average, the calculation is straightforward using the expressions of the averaged elasticity tensors established in the previous section. Velocities are given for an arbitrary direction of the wave propagation, afterwards we will focus on the case of a propagation along the vertical direction **e**_3_ (§5). This is because our motivation comes from application to sonic logging measurements in ice cores, as previously commented.

As a warm up, the case of propagation in single crystals is first considered; this also allows for a validation of our expressions in equations (3.6) and (3.7). Indeed, these are given as a function of the longitudinal angle *φ* of the *c*-axis; with a wave propagation along the vertical direction, the problem becomes invariant by rotation around **e**_3_ so that the wave velocities have to be found independent of *φ*. Then, the propagation in polycrystals using ensemble averages is briefly recalled and the wave velocities are derived.

### (a) The case of propagation in a single crystal

#### (i) Exact expressions of the velocities for single crystals

The propagation of monochromatic waves of frequency *ω* in single crystals is described by the wave equation

4.1 $$\rho \omega^{2}u_{a}+c_{abcd}\frac{\partial^{2}}{\partial x_{b}\partial x_{c}}u_{d}=0.$$

In our case, the problem will be solved considering the wave propagating along the vertical **e**_3_ direction for a *c*-axis having arbitrary direction (thus, without loss of generality at this stage). This approach slightly differs from previous ones where the elasticity tensor is written in the crystal frame (${\text{e}}_{1}^{0},{\text{e}}_{2}^{0},{\text{e}}_{3}^{0}$ associated with the principal directions of anisotropy of the crystal) but where an arbitrary direction of the wave propagation is considered (see [34]; see also [35], which corrects a misprint in the former reference). Obviously, the two approaches are equivalent, and, when propagation in a single crystal only is needed, this latter approach is simpler.

Waves propagating along **e**_3_ correspond to *u*_*a*_=*U*_*a*_ e^i*kx*_3_^, with *k* the wavenumber, and equation (4.1) simplifies to

4.2 $$\left(\begin{array}{ccc} \rho \omega^{2}-k^{2}c_{1331} & -k^{2}c_{1332} & -k^{2}c_{1333}\\ -k^{2}c_{2331} & \rho \omega^{2}-k^{2}c_{2332} & -k^{2}c_{2333}\\ -k^{2}c_{3331} & -k^{2}c_{3332} & \rho \omega^{2}-k^{2}c_{3333} \end{array}\right)\left(\begin{array}{c} U_{1}\\ U_{2}\\ U_{3} \end{array}\right)=0,$$

which admits a non-zero solution for (*U*_1_,*U*_2_,*U*_3_) if the discriminant of the matrix vanishes, leading to a dispersion relation *D*(*ω*,*k*)=0. The dispersion relation admits in general three solutions for the frequencey *ω* as a function of wavenumber *k*. They correspond to three eigenvectors: one longitudinal wave and two transverse waves. One obtains the dispersion relation in the form of a Christoffel equation

4.3 $$\left|\begin{array}{ccc} \rho \omega^{2}-k^{2}C_{55} & -k^{2}C_{45} & -k^{2}C_{35}\\ -k^{2}C_{45} & \rho \omega^{2}-k^{2}C_{44} & -k^{2}C_{34}\\ -k^{2}C_{35} & -k^{2}C_{34} & \rho \omega^{2}-k^{2}C_{33} \end{array}\right|=0,$$

with C, the Voigt matrix, associated with the elasticity tensor *c*_*abcd*_, in equations (3.6) and (3.7). Obviously, for a single crystal, the use of *φ* is useless as the problem is invariant by rotation around **e**_3_ and this has been done in [34]. We have checked that the solutions of the dispersion relation do not depend on *φ* (this has been done numerically by solving the eigenvalue problem associated with equation (4.3) for various *φ*-values and the eigenvalues are found to be independent of *φ*, as expected). For simplicity, we report below the result for *φ*=0, where the coefficients *C*_*ii*_, *i*=3,4,5 and *C*_34_, *C*_45_, *C*_35_ needed in the dispersion relation (equation (4.3)), have the simpler form (from equations (3.6) and (3.7))

4.4 $$\left.\begin{array}{rl} C_{33} & =A\text{s}\theta^{4}+2(2L+F)\text{s}\theta^{2}\text{c}\theta^{2}+C\text{c}\theta^{4},\\ C_{44} & =L\text{c}\theta^{2}+N\text{s}\theta^{2},\\ C_{55} & =(A+C-2F)\text{s}\theta^{2}\text{c}\theta^{2}+L{\left(\text{c}\theta^{2}-\text{s}\theta^{2}\right)}^{2},\\ C_{35} & =-\text{s}\theta \text{c}\theta [A\text{s}\theta^{2}-C\text{c}\theta^{2}+(2L+F)(1-2\text{s}\theta^{2})], \end{array}\right\}$$

and *C*_34_=*C*_45_=0. The discriminant simplifies to

4.5 $$[\rho \omega^{2}-k^{2}C_{44}][(\rho \omega^{2}-k^{2}C_{55})(\rho \omega^{2}-k^{2}C_{33})+k^{4}C_{35}^{2}]=0.$$

The wavenumber *k*^2^=*ρω*^2^/*C*_44_ corresponds to a non-zero eigenmode (0,*U*_2_,0) associated with the transverse SH-wave perpendicular to the propagation plane (**e**_3_,**c**). The two other wavenumbers satisfy a second-order equation (vanishing second term in equation (4.5)), and they correspond to the longitudinal wave P and the transverse wave SV that are coupled when propagating. The associated velocities are

4.6 $$\left.\begin{array}{rl} \rho V_{\mathrm{SH}}^{2} & =L\cos^{2}\theta +N\sin^{2}\theta ,\\ \rho V_{\mathrm{SV}}^{2} & =\frac{1}{2}[C+L+(A-C)\sin^{2}\theta -\sqrt{D}],\\ \rho V_{P}^{2} & =\frac{1}{2}[C+L+(A-C)\sin^{2}\theta +\sqrt{D}],\\ \;\text{with}\;\;D & \equiv [A\text{s}\theta^{2}-C\text{c}\theta^{2}][A\text{s}\theta^{2}-C\text{c}\theta^{2}+2L(\text{c}\theta^{2}-\text{s}\theta^{2})]+4\text{s}\theta^{2}\text{c}\theta^{2}(F^{2}+2FL)+L^{2}, \end{array}\right\}$$

in agreement with [34], eqns A(10) and A(11).

#### (ii) Approximate expressions: the case of an ice single crystal

**Figure 5.**
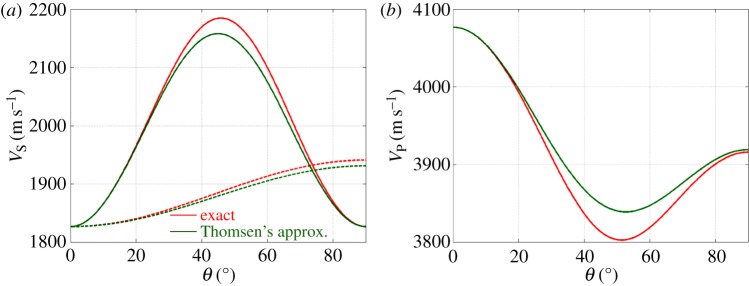
Elastic wave velocities as a function of the $\theta =(\hat{\text{k},\text{c}})$-angle in an ice single crystal. (*a*) PlainQ1 lines *V*_SV_ and dotted lines *V*_SH_; (*b*) *V*_P_. In both (*a*,*b*), red curves refer to the exact values (equations (4.6)), and green curves refer to Thomsen's approximation (equations (4.7)).

The variations of the wave velocities as a function of *θ* (equations (4.6)) are shown in figure 5 (with values from (1.1)).

As ice single crystals are only weakly anisotropic, approximate expressions for the velocities are often used. In 1986, Thomsen [35] remarked that a common approximation is to neglect the anisotropy because ‘the mathematical equations for anisotropic wave propagation are algebraically daunting’ and he proposed a simplified version, which is commonly used in the context of geophysics. They are

4.7 $$\left.\begin{array}{rl} V_{P}^{\mathrm{Th}} & =\sqrt{\frac{C}{\rho }}[1+\delta \sin^{2}\theta \cos^{2}\theta +\beta \sin^{4}\theta ],\\ V_{\mathrm{SV}}^{\mathrm{Th}} & =\sqrt{\frac{L}{\rho }}\left[1+\frac{C}{L}(\beta -\delta )\sin^{2}\theta \cos^{2}\theta \right],\;V_{\mathrm{SH}}^{\mathrm{Th}}=\sqrt{\frac{L}{\rho }}[1+\gamma \sin^{2}\theta ], \end{array}\right\}$$

with

4.8 $$\beta \equiv \frac{A-C}{2C},\;\delta \equiv \frac{{\left(F+L\right)}^{2}-{\left(C-L\right)}^{2}}{2C(C-L)}\;\mathrm{and}\;\gamma \equiv \frac{N-L}{2N}$$

being small parameters (*β*∼4%, *γ*∼7% and *δ*∼20%), and the reference velocities being $V_{P}^{\mathrm{Th}}∼\sqrt{C/\rho }=4077\;m\;s^{-1}$ and $V_{S}^{\mathrm{Th}}∼\sqrt{L/\rho }=1827\;m\;s^{-1}$. These approximate velocities are also reported in figure 5 for comparison with the exact ones. The errors in Thomsen's approximations are indeed small: 0.5% for an SP-wave, 0.3% for SH-waves and 0.4% for P-waves.

### (b) Effective elastic velocities in textured polycrystals

When an ensemble of grains with different anisotropy directions is considered, it is difficult and not useful to describe wave propagation through a single, particular, distribution. Rather, as discussed in the Introduction, ensemble averages can be considered, leading to a description of the properties averaged over all possible realizations of the disorder. In this paper, we shall write the elastic constants as a sum of an average plus deviations from that average: *c*_*abcd*_=〈*c*_*abcd*_〉+*δc*_*abcd*_, so the wave propagation in a given realization reads

4.9 $$\rho \omega^{2}u_{a}+\langle c_{abcd}\rangle \frac{\partial^{2}}{\partial x_{b}\partial x_{c}}u_{d}=-\frac{\partial }{\partial x_{b}}\delta c_{abcd}\frac{\partial }{\partial x_{c}}u_{d},$$

where *δc*_*abcd*_ is the local deviation of the elasticity tensor from its average value, and it is assumed to be small; that is, if *c* and *δc* are the typical magnitudes of 〈*c*_*abcd*_〉 and *δc*_*abcd*_, we define the small parameter *ϵ*=*δc*/*c*. Equation (4.9) is the wave equation through a medium with average properties given by the effective stiffness tensor 〈*c*_*abcd*_〉 and submitted to a ‘potential’ *V*_*ad*_=(∂/∂*x*_*b*_)*δc*_*abcd*_(∂/∂*x*_*c*_) which reflects the fluctuations of the real medium with respect to the averaged one. If *ϵ*≪1, the wave propagation is mainly determined by the simplified equation

4.10 $$\rho \omega^{2}u_{a}+\langle c_{abcd}\rangle \frac{\partial^{2}}{\partial x_{b}\partial x_{c}}u_{d}=0,$$

and any particular realization of the propagation will be close to this average, up to *ϵ*^2^. Indeed, because 〈*δc*_*abcd*_〉=0 by construction, the potential *V*_*ad*_ has no contribution at first order in *ϵ*. At second and higher orders, it produces a hierarchy of corrections, including wave attenuation and backscattering (e.g. [1,15]). The successive iterations correspond to the successive approximations of the Dyson equation. Below, the zeroth-order approximation is considered and the accuracy of this zeroth-order will be discussed in more detail in the case of ice polycrystals in §5*c*.

**Figure 6.**
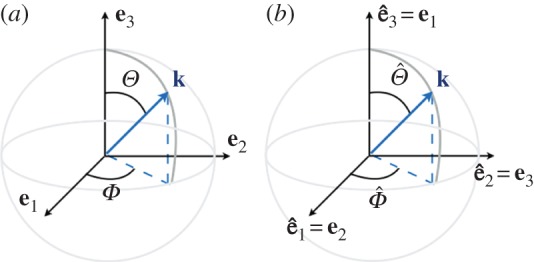
Orientation of the **k**-vector (*a*) as used for the cluster and partial girdle fabrics with (*Θ*,*Φ*), and (*b*) as used for the thick girdle fabric with $\left(\hat{\Theta },\hat{\Phi }\right)$.

The elasticity tensor 〈*c*_*ijkl*_〉 has been expressed in the previous section using a frame where the direction **e**_1_,**e**_2_ or **e**_3_ is an axis of symmetry. To get the dispersion relation in a general case, one has to consider now an arbitrary direction of the wave propagation given by the wave vector **k**=(*k*_1_,*k*_2_,*k*_3_) (and we defined **k** using the co-latitude and longitudinal angles *Θ* and *Φ*; figure 6). Our Voigt matrices for the considered cluster and girdle fabrics are at least orthorhombic (equation (3.15)), and we restrict the dispersion relations to this symmetry (which includes the hexagonal symmetry). Within this symmetry and looking for a wave *u*_*a*_=*U*_*a*_ e^i(*k*_1_*x*_1_+*k*_2_*x*_2_+*k*_3_*x*_3_)^, the dispersion relation takes the form,

4.11 $$\left|\begin{array}{ccc} \rho \omega^{2}-A_{165} & -k_{1}k_{2}B_{126} & -k_{1}k_{3}B_{135}\\ -k_{1}k_{2}B_{126} & \rho \omega^{2}-A_{624} & -k_{2}k_{3}B_{234}\\ -k_{1}k_{3}B_{135} & -k_{2}k_{3}B_{234} & \rho \omega^{2}-A_{543} \end{array}\right|=0,$$

where we have defined $A_{ijk}\equiv [k_{1}^{2}\langle C_{ii}\rangle +k_{2}^{2}\langle C_{jj}\rangle +k_{3}^{2}\langle C_{kk}\rangle ]$ and *B*_*ijk*_≡[〈*C*_*ij*_〉+〈*C*_*kk*_〉]. Depending on the fabric, we will derive in the following sections the corresponding velocities owing to the Voigt matrices previously calculated (equations (3.10), (3.16) and (3.22)).

#### (iii) Cluster fabric

The cluster fabric has hexagonal symmetry, being isotropic in the (**e**_1_,**e**_2_) plane. It is thus sufficient to define the angle *Θ* that **k** forms with **e**_3_. With $k_{1}=0,k_{2}=k\sin\Theta \;\mathrm{and}\;k_{3}=k\cos\Theta$, equation (4.11) simplifies to

4.12 $$\left|\begin{array}{ccc} \rho \omega^{2}-[\langle C_{66}\rangle \text{s}\Theta^{2}+\langle C_{44}\rangle \text{c}\Theta^{2}]k^{2} & 0 & 0\\ 0 & \rho \omega^{2}-[\langle C_{11}\rangle \text{s}\Theta^{2}+\langle C_{44}\rangle \text{c}\Theta^{2}]k^{2} & -\text{s}\Theta \text{c}\Theta [\langle C_{13}\rangle +\langle C_{44}\rangle ]k^{2}\\ 0 & -\text{s}\Theta \text{c}\Theta [\langle C_{13}\rangle +\langle C_{44}\rangle ]k^{2} & \rho \omega^{2}-[\langle C_{44}\rangle \text{s}\Theta^{2}+\langle C_{33}\rangle \text{c}\Theta^{2}]k^{2} \end{array}\right|=0$$

(with s*Θ*, c*Θ* denoting sin⁡Θ, cos⁡Θ), and with the Voigt matrix defined in equation (3.10), with 〈*C*_55_〉=〈*C*_44_〉. We deduce the velocities (the plane of incidence being (**e**_2_,**e**_3_))

4.13 $$\mathrm{cluster}\left\{\begin{array}{rl} \rho V_{\mathrm{SH}}^{2} & =\langle C_{44}\rangle \cos^{2}\Theta +\langle C_{66}\rangle \sin^{2}\Theta ,\\ \rho V_{\mathrm{SV}}^{2} & =\frac{1}{2}[\langle C_{33}\rangle +\langle C_{44}\rangle +(\langle C_{11}\rangle -\langle C_{33}\rangle )\sin^{2}\Theta -\sqrt{D}],\\ \rho V_{P}^{2} & =\frac{1}{2}[\langle C_{33}\rangle +\langle C_{44}\rangle +(\langle C_{11}\rangle -\langle C_{33}\rangle )\sin^{2}\Theta +\sqrt{D}],\\ \text{with}\;D & \equiv [\langle C_{11}\rangle \text{s}\Theta^{2}-\langle C_{33}\rangle \text{c}\Theta^{2}][\langle C_{11}\rangle \text{s}\Theta^{2}-\langle C_{33}\rangle \text{c}\Theta^{2}\\ & \;+2\langle C_{44}\rangle (\text{c}\Theta^{2}-\text{s}\Theta^{2})]+4\text{s}\Theta^{2}\text{c}\Theta^{2}({\left\langle C_{13}\right\rangle }^{2}+2\langle C_{13}\rangle \langle C_{44}\rangle )+{\left\langle C_{44}\right\rangle }^{2}. \end{array}\right.$$

Obviously, these expressions are analogous to the expressions found for single crystals in equations (4.6) as the hexagonal symmetry is the same. Also, the limiting cases are the direct consequences of the limiting cases for the Voigt matrix: for *θ*_0_=*π*/2 in equations (3.10) (leading to equations (3.12)), the polycrystal is isotropic with shear and compressional velocities simplifying to

4.14 $$\text{isotropic case}\;\left\{\begin{array}{rl} V_{P}^{\text{iso}} & =\sqrt{\frac{\left[8A+3C+4(2L+F)\right]}{15\rho }},\\ V_{S}^{\text{iso}} & =\sqrt{\frac{\left[A+C-2F+6L+5N\right]}{15\rho }}. \end{array}\right.$$

**Figure 7.**
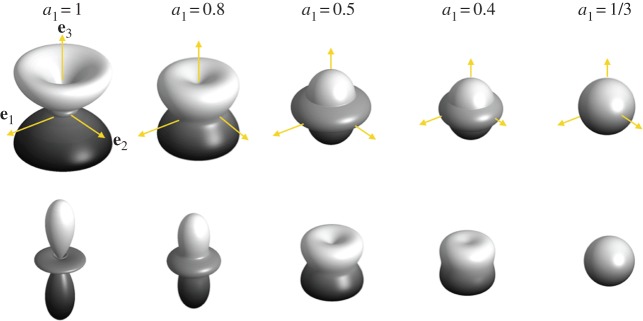
Directional dependence of the S- and P-velocities (as a function of the direction of propagation of the wave), *V*_S_(*Θ*,*Φ*) (the highest value is considered) and *V*_P_(*Θ*,*Φ*), for cluster fabrics. The distance between zero and any point on the surfaces is equal to the velocity $V_{w}-V_{w}^{\mathrm{ref}}$ in that direction, with *w*=*S*,*P*; *V*^ref^_*w*_ is a value of the velocity chosen to illustrate the anisotropy (1800 m s^−1^ for *V*_S_ velocity and 3750 m s^−1^ for *V*_P_).

These expressions of the velocities in the isotropic case do not coincide with the leading order in Thomsen's approximation [35] (equations (4.7)), with *β*=*δ*=*γ*≃0 (leading to $V_{P}^{\mathrm{Th}}≃\sqrt{C/\rho }$ and $V_{S}^{\mathrm{Th}}≃\sqrt{L/\rho }$). This is because Thomsen defines the anisotropy of polycrystals with respect to the isotropy of a single crystal (*a*_1_=1), while the isotropy leading to the velocities above refers to an isotropic polycrystal with *a*_1_=1/3; this will be discussed further in §5*a*. The dependence of the largest S-wave velocity and of the P-wave velocities on the **k**-direction of propagation is illustrated in figure 7 for different *a*_1_ values. For a value of *a*_1_ between 1 and 1/3, the range of the *Θ*-dependent velocities decreases to a single value in the isotropic case. For *a*_1_=1, we recover the angular dependence of single crystals (figure 5), with *V*_S_∈[1831.8,2177.5] m s^−1^ and *V*_P_∈[3775.7,4044.5] m s^−1^. For the isotropic case, *a*_1_=1/3, we get *V*^iso^_S_=1956.1 m s^−1^ and *V*^iso^_P_=3847.7 m s^−1^.

#### (iv) Girdle fabrics

**Figure 8.**
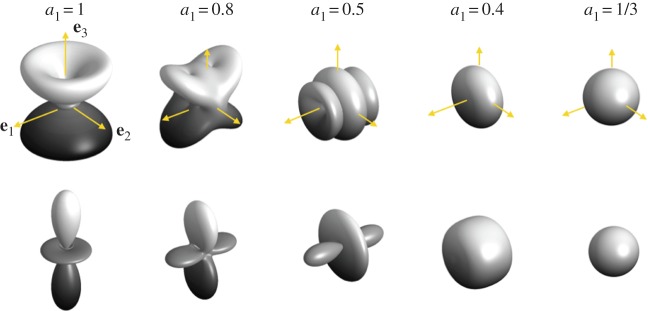
Directional dependence of the S- and P-velocities, *V*_S_(*Θ*,*Φ*) (the highest value is considered) and *V*_P_(*Θ*,*Φ*), for girdle fabrics (partial girdle for *a*_1_≥0.5 and thick girdle for *a*_1_≥0.5). Same representation as in figure 7.

The case of partial girdles does not lead to substantial simplifications of the dispersion relation, as one has to account in general for the three components of **k**. The dispersion relation has to be solved for a given *Θ* and *Φ*, the co-latitude and longitudinal angles of **k** in (**e**_1_,**e**_2_,**e**_3_), and no tractable expression can be given. Closed forms of the velocities will be considered in §5 for waves propagating along the vertical direction. Nevertheless, it is straightforward to solve the dispersion relation, for instance by determining the eigenvalues of the Voigt matrix divided by *ρ*, with *V*^2^=*ω*^2^/*k*^2^. This has been done numerically and the results for *a*_1_=0.8 and 0.5 (perfect girdle with symmetry by rotation around **e**_1_) are reported in figure 8.

For a thick girdle, the symmetry is hexagonal with isotropic behaviour in the (**e**_2_,**e**_3_) plane. Using the notations in figure 6*b*, we can use directly the result of equations (4.16), with the Voigt matrix $\hat{C}$. Next, using the correspondences in equations (3.20), we get, for *k*_2_=0, $k_{1}=k\cos\hat{\Theta }$ and $k_{3}=k\sin\hat{\Theta }$ (that is, for $\hat{\Phi }=\pi /2$), the dispersion relation

4.15 $$\left|\begin{array}{ccc} \rho \omega^{2}-[\langle C_{11}\rangle \text{c}{\hat{\Theta }}^{2}+\langle C_{55}\rangle \text{s}{\hat{\Theta }}^{2}]k^{2} & 0 & -\text{s}\hat{\Theta }\text{c}\hat{\Theta }[\langle C_{12}\rangle +\langle C_{55}\rangle ]k^{2}\\ 0 & \rho \omega^{2}-[\langle C_{44}\rangle \text{s}{\hat{\Theta }}^{2}+\langle C_{55}\rangle \text{c}{\hat{\Theta }}^{2}]k^{2} & 0\\ -\text{s}\hat{\Theta }\text{c}\hat{\Theta }[\langle C_{12}\rangle +\langle C_{55}\rangle ]k^{2} & 0 & \rho \omega^{2}-[\langle C_{22}\rangle \text{s}\Theta^{2}+\langle C_{55}\rangle \text{c}\Theta^{2}]k^{2} \end{array}\right|=0,$$

where we have used that 〈*C*_33_〉=〈*C*_22_〉, 〈*C*_66_〉=〈*C*_55_〉 and 〈*C*_13_〉=〈*C*_12_〉 (equation (3.21)). We deduce the velocities

4.16 $$\left.\begin{array}{rl} \rho V_{\mathrm{SH}}^{2} & =\langle C_{55}\rangle \cos^{2}\hat{\theta }+\langle C_{44}\rangle \sin^{2}\hat{\theta },\\ \rho V_{\mathrm{SV}}^{2} & =\frac{1}{2}[\langle C_{11}\rangle +\langle C_{55}\rangle +(\langle C_{22}\rangle -\langle C_{11}\rangle )\sin^{2}\hat{\theta }-\sqrt{D}]\\ \;\text{and}\;\;\rho V_{P}^{2} & =\frac{1}{2}[\langle C_{11}\rangle +\langle C_{66}\rangle +(\langle C_{22}\rangle -\langle C_{11}\rangle )\sin^{2}\hat{\theta }+\sqrt{D}], \end{array}\right\}$$

with *D*≡[〈*C*_22_〉s*Θ*^2^−〈*C*_11_〉c*Θ*^2^][〈*C*_22_〉s*Θ*^2^−〈*C*_11_〉c*Θ*^2^+2〈*C*_55_〉(c*Θ*^2^−s*Θ*^2^)]+4s*Θ*^2^c*Θ*^2^(〈*C*_12_〉^2^+2〈*C*_12_〉〈*C*_55_〉)+〈*C*_55_〉^2^.

## 5. Comments and comparison with previous work on ice polycrystals

In this section, we focus more specifically on the application to ice fabric characterization using a sonic log of the deep boreholes, as tested during January 2011 at Dome C, East Antarctica, by Gusmeroli *et al*. [24]. To the best of our knowledge, this is only the second *in situ* measurement campaign, the first one being the deep drill hole at Byrd Station, Antarctica, during the 1969–1970 field seasons by Bentley [23]. In between, laboratory measurements of the velocity have been performed using core samples: in the Greenland Ice Sheet Project II (GISP2) deep core [36,37] and in the Dye 3, Greenland, deep ice core [38,39]. Except in [24], these studies have confirmed qualitatively, but not quantitatively, the sensitivity of the elastic waves to the degree of anisotropy of cluster fabrics (which are the dominant fabrics at Dye 3, GISP2 and Dome C). The exception in [24] is the quantitative inspection that is proposed, by means of an inversion procedure and by means of a comparison between the *in situ* measurements of the velocities and values of the velocities deduced from the measures of *c*-axis orientations in thin sections of core samples. The logs as used in *in situ* measurements provide the time of flight between two receivers located on a vertical logger which can be used to obtain the S- and P-wave velocities. Then, an inverse procedure has to be used to obtain information on the fabric in the interrogated zone. This procedure raises several questions that we will address in this section. They are (i) a comparison of our expressions of the velocities with the semi-empirical determination of the elastic velocities, as proposed by Bennett [21] for clustered fabrics because of their averred successful comparisons with experiments. We did not find an expression of the velocities for girdle fabrics in the previous literature that could be used for comparison with our present result. (ii) A discussion on the variation of the velocities from one fabric to another; and within a given fabric, how the velocities change with the degree of anisotropy. It is, of course, necessary that the velocity change that a purported fabric or anisotropy induces is quantified and that this change is larger than the accuracy of the experimental data, if it is to be used as initial data in an inverse problem. (iii) In connection with the previous question, the modelling presented in this paper has to be sufficiently accurate if it is to be used in the inverse process. Thus, the accuracy of the zeroth-order Dyson equation that we employ is addressed. As a prerequisite, note that the sonic measurements are performed at about 20 KHz, so the wavelength in ice is typically 10–20 cm, much larger than the grain size (1 mm–1 cm).

### (a) Velocities for vertical wave propagation in an ice polycrystal

As discussed in §1, we have chosen the **e**_3_ axis to coincide with the vertical direction, a convenient choice to analyse sonic logging measurements of antarctic ice. As a first step, we report below the expression of the velocities for propagation of the wave along the vertical **e**_3_ axis. In this case, the expressions from equations (4.11) simplify to $V_{P}=\sqrt{C_{33}/\rho }$, $V_{S1}=\sqrt{C_{44}/\rho }$ and $V_{S2}=\sqrt{C_{55}/\rho }$ (to see this, take *k*_1_=*k*_2_=0 in equations (4.11); it leads to the determinant of a diagonal matrix). Here, we use *V*_S1_ and *V*_S2_ with *V*_S1_ the largest velocity (when different, the two S-waves cannot be identified *a priori* and the term of ‘quasi-’ S-wave is preferred, e.g. [40]). For cluster fabrics, from equations (3.10) (or using *Θ*=0 in equations (4.16) and $\sqrt{D}=\langle C_{33}\rangle -\langle C_{44}\rangle >0$), we get

5.1 $$\mathrm{cluster}\left\{\begin{array}{l} V_{S}=\sqrt{\frac{\left(L+N\right)}{2\rho }+\frac{\left[2(A+C)-4F-3L-5N\right]}{30\rho }X-\frac{\left[(A+C)-2(2L+F)\right]}{10\rho }Y}\\ V_{P}=\sqrt{\frac{A}{\rho }+\frac{\left[-7A+3C+4(2L+F)\right]}{15\rho }X+\frac{\left[A+C-2(2L+F)\right]}{5\rho }Y}, \end{array}\right.$$

with, as previously, $X\equiv 1+\cos\theta_{0}+\cos^{2}\theta_{0}$ and $Y\equiv \cos^{3}\theta_{0}+\cos^{4}\theta_{0}$.

For partial girdle fabrics (P.G.), with *C*_33_, *C*_44_ and *C*_55_ given by equations (3.16), we get

5.2 $$P.G.\left\{\begin{array}{l} V_{P}=\sqrt{\frac{\left[3(A+C)+2(2L+F)\right]}{8\rho }-\frac{\left[A-C\right]}{2\rho }\text{sinc}2\theta_{0}+\frac{\left[A+C-2(2L+F)\right]}{8\rho }\text{sinc}4\theta_{0}},\\ V_{S1}=\sqrt{\frac{\left[A+C-2F+4L\right]}{8\rho }-\frac{\left[A+C-2(F+2L)\right]}{8\rho }\text{sinc}4\theta_{0}},\\ V_{S2}=\sqrt{\frac{\left[L+N\right]}{2\rho }+\frac{\left[L-N\right]}{2\rho }\text{sinc}2\theta_{0}}. \end{array}\right.$$

For the thick girdle (T.G.), with *C*_33_, *C*_44_ and *C*_55_ being given in equations (3.22), we have

5.3 $$T.G.\left\{\begin{array}{l} V_{P}=\sqrt{\frac{\left[3(A+C)+2(2L+F)\right]}{8\rho }+\frac{\left[A-3C+2(2L+F)\right]}{12\rho }\sin^{2}\xi_{0}+\frac{3[A+C-2(2L+F)]}{40\rho }\sin^{4}\xi_{0}},\\ V_{S1}=\sqrt{\frac{\left[A+C-2F+4L\right]}{8\rho }-\frac{\left[A+C-2(2N+F)\right]}{12\rho }\sin^{2}\xi_{0}+\frac{\left[A+C-2(2L+F)\right]}{40\rho }\sin^{4}\xi_{0}},\\ V_{S2}=\sqrt{\frac{\left[L+N\right]}{2\rho }+\frac{\left[A+C-2F-3L-N\right]}{6}\sin^{2}\xi_{0}-\frac{\left[A+C-2(2L+F)\right]}{10\rho }\sin^{4}\xi_{0}}. \end{array}\right.$$

**Figure 9.**
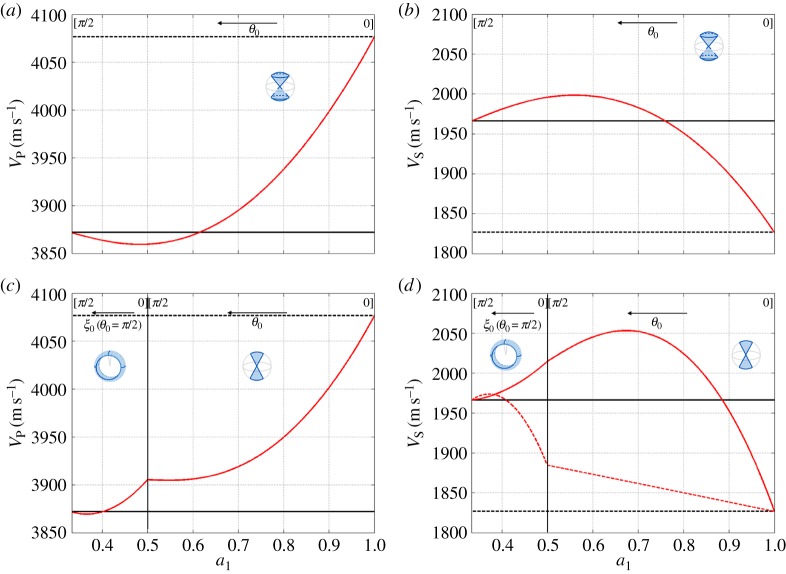
Variation of the compressional P-wave velocity, *V*_P_, and shear S-wave velocities, *V*_S1_ and *V*_S2_, as a function of the degree of anisotropy of the polycrystal, for: (*a*,*b*) a cluster fabric, from equations (5.1) with equation (2.6) and (*c*,*d*) for a girdle fabric, from equations (5.2) with (2.11) and (5.3) with (2.17).

Figure 9 shows the variation of the velocities as a function of the degree of anisotropy of the polycrystal, measured with *a*_1_ (defined in equations (2.6), (2.11) and (2.17)). For comparison, we also present the velocities for the isotropic single crystal, which is the leading order in the Thomsen's approximation (equations (4.7) with *β*=*δ*=*γ*≃0), and the velocities that correspond to an ice polycrystal with randomly oriented *c*-axes (equations (4.14)). As expected, these two limits correspond to velocities obtained at *a*_1_=1 (single maximum) and *a*_1_=1/3 (isotropic polycrystal), respectively. It is often tempting to neglect the anisotropy of an ice polycrystal and we report here the relative errors in the velocities when using this approximation—when using single-crystal isotropy (Thomsen's leading order): for the cluster 3.5% and 5.7% (P- and S-waves) and for the girdles 3.8%, 7.7% and 3.7% (for P-, SV- and SH-waves, respectively); when using polycrystal isotropy: for the cluster 1.9% and 2.5% (P- and S-waves) and for the girdles 1.4%, 2.3% and 3.7% (for P-, SV- and SH-waves, respectively); thus, polycrystal isotropy is a better approximation.

Next, we come back to the range of variation of the velocities with respect to the degree of anisotropy of the fabrics. We report the relative variation 2(*V*_max_−*V*_min_)/(*V*_max_+*V*_min_) for each fabric

5.4 $$\left.\begin{array}{l} \mathrm{forclusters}:\;S-9.0\%,\;P-5.5\%.\\ \mathrm{forgirdle}:\;S{\$}_{1}\$-11.7\%,\;S{\$}_{2}\$-7.7\%,\;P-5.2\%. \end{array}\right\}$$

These ranges of variation are small, because an ice single crystal is weakly anisotropic; with the same definitions of the velocity relative variation (for ice single crystal: SV-17.8%, SH-6.1%, P-7.0%). Thus, in all the steps of the measurements and of the inversion process, the errors have to be smaller than, say, 1%.

### (b) Comparison with previous results

#### (i) Remark on the importance of the *φ*-average

As discussed in the Introduction, a common approach to derive the effective velocities in a polycrystal consists in averaging the ‘time of flight’, that is,

5.5 $$V_{P}^{-1}=\langle {\left(V_{P}\right)}^{-1}\rangle \;\mathrm{and}\;V_{S.}^{-1}=\langle {\left(V_{S.}\right)}^{-1}\rangle ,$$

and, in most of the studies on clusters (as used in [24]), the average is done on the colatitude *θ* only, that is, the angle between the *c*-axis and the direction **e**_3_ of the wave propagation, and not on the longitude *φ*. At first sight, this approach appears reasonable. However, there are two pitfalls. (i) The times of flight are calculated for each S- or P-wave separately, assuming that the wave energies originally distributed into S- and P-waves remain unchanged; in other words, the re-repartition of the energy between SH-, SV- and P-waves at each grain boundary by mode conversion is disregarded. (ii) More seriously in our opinion, it is assumed that an ensemble of grains with the same *θ* but different *φ*-value behaves as a homogeneous medium, which is clearly not the case. Although this crystallographic arrangement is artificial, it allows us to exemplify the error made when ignoring the *φ*-dependence. Let us first remark that the structure resulting from such an arrangement has an anisotropy with hexagonal symmetry (effective *c*-axis along **e**_3_), thus the two shear velocities are the same for waves propagating along the **e**_3_-axis. From appendix A, the velocities for grains with the same *θ* and different *φ*-values are equivalent to an HTI structure (with symmetry around **e**_3_), and, from equations (3.7) and (A 3), we get

5.6 $$\mathrm{all}\;c\;\mathrm{with}\;\mathrm{same}\;\theta \left\{\begin{array}{l} V_{P}^{2}(\theta )=\frac{1}{\rho }[A\text{s}\theta^{4}+2(2L+F)\text{s}\theta^{2}\text{c}\theta^{2}+C\text{c}\theta^{4}],\\ V_{S}^{2}(\theta )=\frac{1}{2\rho }[(A+C-2F)\text{s}\theta^{2}\text{c}\theta^{2}+L(4\text{s}\theta^{4}-5\text{s}\theta^{2}+2)+N\text{s}\theta^{2}], \end{array}\right.$$

and the S-velocities are correctly found to be the same. On the contrary, if the average is performed on *θ* only (and the average in this case consists in selecting a unique value of *θ* by a delta function), equations (5.5) predict that the ensemble of grains behaves as a single crystal with SH- and SV-wave velocities being given by equations (4.6), and this cannot be the case. Incidentally, note that not only the *V*_S_(*θ*) but also *V*_P_(*θ*) is false when using equations (5.5) (see *V*_P_ in equations (4.6) and (5.6)).

#### (ii) Bennett's prediction

Alternatively to the slowness average, Bennett [21] provides a prediction for *V*_P_ and *V*_S_ based on acoustic measurements. The details of the method are given in [21], ch. 5 and in [22], ch. 6 and they are not discussed here. These semi-empirical derivations of the velocities have been shown to be in agreement with experiments so it makes sense to use them as a validation. For a propagation along the **e**_3_-axis, Bennett's velocities read

5.7 $$\left.\begin{array}{rl} {\left(V_{P}^{\mathrm{Be}}\right)}^{2} & ≃\frac{1}{{\left(a_{1}-b_{1}+c_{1}\right)}^{2}}\left[1-\frac{4(8b_{1}-5c_{1})}{15(a_{1}-b_{1}+c_{1})}X+\frac{16b_{1}}{5(a_{1}-b_{1}+c_{1})}Y\right],\\ {\left(V_{S}^{\mathrm{Be}}\right)}^{2} & ≃\frac{1}{a_{3}^{2}}\left[1-\frac{2(5b_{3}-8b_{2})}{15a_{3}}X-\frac{8b_{2}}{5a_{3}}Y\right], \end{array}\right\}$$

where (*X*,*Y*) are the same as used in equations (3.11), and the *a*_*i*_ and *b*_*i*_ are constants given by Bennett's fits: *a*_1_=256.28, *b*_1_=5.92, *c*_1_=5.08, *a*_3_=531.40, *b*_2_=45.37 and *b*_3_=15.94 (in μs m^−1^). Bennett's fits are usually reported in the literature with a different form. For convenience, we have rewritten them in the form of equations (5.7):

5.8 $$\left.\begin{array}{rl} V_{P}^{2} & =V_{P0}^{2}[1-\epsilon_{P}^{X}\;X-\epsilon_{P}^{Y}Y],\\ V_{S}^{2} & =V_{S0}^{2}[1-\epsilon_{S}^{X}\;X-\epsilon_{S}^{Y}Y]. \end{array}\right\}$$

Inspecting equations (5.1) and (5.7), we get the correspondences between our velocities and Bennett's ones. The results of (5.1) are obtained from (5.8) by

5.9 $$\mathrm{from (5.1)}\;\left\{\begin{array}{l} V_{P0}=\sqrt{\frac{A}{\rho }},\;V_{S0}=\sqrt{\frac{\left(L+N\right)}{2\rho }},\\ \epsilon_{P}^{X}=\frac{\left[7A-3C-4(F+2L)\right]}{15A},\;\epsilon_{P}^{Y}=-\frac{\left[A+C-2(2L+F)\right]}{5A},\\ \epsilon_{S}^{X}=-\frac{\left[2(A+C-2F)-3L-5N\right]}{15(L+N)},\;\epsilon_{S}^{Y}=\frac{\left[A+C-2F-4L\right]}{5(L+N)}, \end{array}\right.$$

and the results of (5.7) are obtained from (5.8) by

5.10 $$\mathrm{from (5.7)}\;\left\{\begin{array}{l} V_{P0}=\frac{1}{\left(a_{1}-b_{1}+c_{1}\right)},\;V_{S0}=\frac{1}{a_{3}},\\ \epsilon_{P}^{X}=\frac{4(8b_{1}-5c_{1})}{15(a_{1}-b_{1}+c_{1})},\;\epsilon_{P}^{Y}=-\frac{16b_{1}}{5(a_{1}-b_{1}+c_{1})},\\ \epsilon_{S}^{X}=\frac{2(5b_{3}-8b_{2})}{15a_{3}},\;\epsilon_{S}^{Y}=\frac{8b_{2}}{5a_{3}}. \end{array}\right.$$

**Figure 10.**
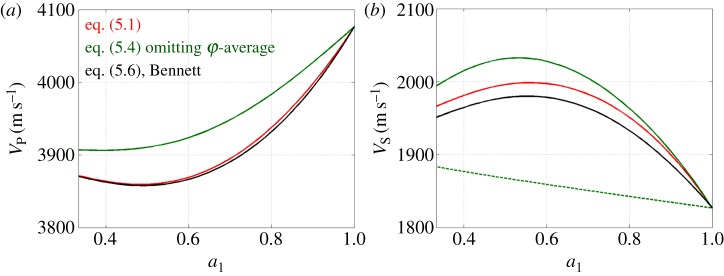
Comparison of the velocities from our expressions (equations (5.1)) (red), Bennett's fits (equations (5.7)) and as used in [24], omitting the *φ*-average. For *V*_S_, this latter calculation leads to two (unphysical) velocities (green solid and dotted lines).

Table 1 reports the values of the coefficients defined in equations (5.8), from equations (5.9) using Bennett's values of the elastic constants (*A*,*L*,*N*,*F*) and density *ρ* given by (1.1), and from equations (5.10) using the values of (*a*_*i*_,*b*_*i*_) given above. The agreement is excellent, although less good for the S-wave than for the P-wave, and this will be analysed in more detail in forthcoming work. The resulting agreement on the velocities is 0.07% for the P-wave and 0.7% for the S-wave, without any adjustment (figure 10) (red and black curves). For completeness, we also report in figure 10 the results obtained from slowness averaging as used in [24], omitting the *φ*-average (equations (5.5)) (green curves). This latter case leads to two different S-wave velocities, which is unphysical as the wave propagates along the symmetry axis. Incidentally, there is a slightly more notable disagreement with Bennett [21], with 0.9% for both the P- and the S-wave velocities (when compared with the highest S-velocity).

**Table 1.** Comparison between Bennett's prediction and our calculation.

coeffs in equations (5.8)	*ϵ*_P,*X*_	*ϵ*_P,*Y*_	*ϵ*_S,*X*_	*ϵ*_S,*Y*_	*V*_P0_	*V*_S0_
from (5.9)	0.0223	−0.0754	−0.0883	0.1627	3.9157 10^3^	1.8848 10^3^
from (5.10)	0.0229	−0.0742	−0.0711	0.1366	3.9148 10^3^	1.8818 10^3^

### (c) Inspecting the accuracy of our prediction

In our study, 〈*c*_*ijkl*_〉 corresponds to anisotropic, or textured, effective media, with orthorhombic or hexagonal symmetry. For ice, *δc* is a small correction as ice single-crystal anisotropy is weak, as previously commented. Thus, the zeroth order of the Dyson equation, which we have used by solving equation (4.10) instead of equation (4.9), is justified but the results are accurate up to *ϵ*^2^=(*δc*/*c*)^2^ only. It is of importance to get an estimate of *ϵ*^2^ as the accuracy of the prediction on the velocities has to be sufficient to resolve the typical variations of the velocities.

**Figure 11.**
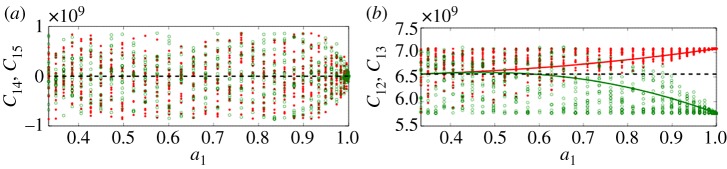
Fluctuations of the *C*_*ij*_ coefficients within actual grains (composed by single crystal ice) whose assembly forms a cluster fabric. Symbols show the *C*_*ij*_-values, for each *a*_1_ value, for 20 different orientations (randomly chosen in [0,*θ*_0_]). (*a*) Two parameters with zero mean, (*b*) two parameters with non-zero mean; 〈*C*_12_〉 and 〈*C*_13_〉 are indicated by solid lines. The dotted line shows $C_{12}^{\mathrm{iso}}=C_{13}^{\mathrm{iso}}$.

**Figure 12.**
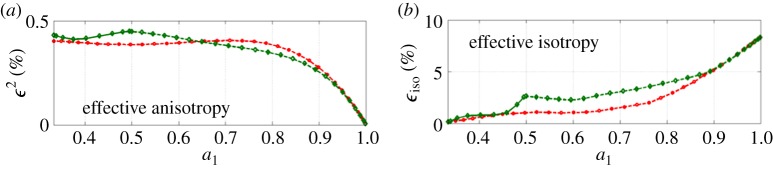
Accuracy of the approximations (*a*) at second-order *ϵ*^2^ when considering an effective anisotropy, equation (4.10) and (*b*) at first-order *ϵ*_iso_ when considering affective isotropy. In red, for clusters and in green for girdles.

This is reported in figures 11 and 12. First, we report typical fluctuations of the *C*_*ij*_ parameters (four terms are shown in figure 11), for a cluster fabric as a function of *a*_1_, that is, for decreasing cone angle *θ*_0_; each point in these plots corresponds to a value of *C*_*ij*_ of one grain with an orientation randomly chosen in [0,*θ*_0_]. The dispersion of the *C*_*ij*_ indicates how far the actual grains are from their effective medium. For all *a*_1_ values, the effective medium has hexagonal symmetry, thus vanishing 〈*C*_14_〉, 〈*C*_15_〉 and they indeed fluctuate with zero mean. For the non-zero terms of the Voigt matrix (*C*_12_ and *C*_13_ are considered), fluctuations occur, with mean values being our 〈*C*_12_〉 and 〈*C*_13_〉; the dotted line shows the value *C*^iso^ (equations (3.12) with $C_{12}^{\mathrm{iso}}=C_{13}^{\mathrm{iso}}$), which appears to be a good estimate of the mean for low *a*_1_ values. Next, we can get an estimate of *ϵ*^2^, the accuracy of our zeroth-order approximation. This is done by calculating numerically the error *ϵ*^2^

5.11 $$\epsilon^{2}=\frac{\left\langle \sum_{i,j}∥C_{ij}-\langle C_{ij}\rangle {∥}^{2}\right\rangle }{\left\langle \sum_{i,j}∥C_{ij}{∥}^{2}\right\rangle },$$

with the averages defined in equations (2.4), (2.8) and (2.14). Results are reported in figure 12*a*; the error appears to be less than 0.5% for any degree of anisotropy *a*_1_ and for both fabrics.

We also report (figure 12*b*) the error *ϵ*_iso_, which defines the accuracy of the results if effective isotropy is assumed,

5.12 $$\epsilon_{\mathrm{iso}}=\frac{\left\langle \sum_{i,j}∥C_{ij}-C_{ij}^{\mathrm{iso}}∥\right\rangle }{\left\langle \sum_{i,j}∥C_{ij}∥\right\rangle },$$

and the error is here of first order, as $\left\langle C_{ij}-C_{ij}^{\mathrm{iso}}\right\rangle$ does not vanish, except for *a*_1_=1/3. As expected, the error is significantly higher and it increases with the degree of anisotropy *a*_1_ up to about 10%.

## 6. Concluding remarks

We have derived closed forms of the elasticity tensors and of the elastic wave velocities for ice textured polycrystals with cluster and girdle fabrics. The prediction is accurate up to the second order in the local deviation of the elasticity parameters from their average values. This is justified for weak anisotropic single crystals or for fabrics with concentrated *c*-axes, that is, in general a system with a low variability in the elasticity tensor of the grains. Motivated by measurements in deep ice cores, we have inspected this small variability case in more detail, revealing an accuracy better than 0.5%. This suggests that velocity measurements can confidently be used to characterize ice fabrics. In a forthcoming publication, we will address the specifics related to a more complete inspection of this problem. More generally, extensions of this study are at least twofold. (i) As few theoretical studies of the effects of texture on backscattering and attenuation have been conducted, it should be interesting to iterate the Dyson equation using the potential defined in equation (4.9) to quantify such effects. (ii) We have used simple ODFs which are based on the observation of the textures; other ODFs, as proposed in [41,27], use two or three tuneable parameters, being related to the fabric evolution under external stresses (due to the gravity and to possible ice flows). Being more involved, it does not allow for analytical calculations, but it should be used to quantify the sensitivity of the velocities on the form of the ODF.

## Data accessibility

This work does not have any experimental data.

## Appendix A. Effective stiffness tensor for a cluster fabric

**(a) The average over *φ***

This first average over *φ* corresponds to a *c*-axis rotating about the vertical axis **e**_3_ with a constant angle *θ*. To omit multiple notations, we note *C*_*ij*_(*θ*) is the resulting average (〈*C*_*ij*_〉 is the final result). With *φ*∈[0,2*π*], we thus have

A 1 $$C_{ij}(\varphi ,\theta )\to C_{ij}(\theta )\equiv \frac{1}{2\pi }\int_{0}^{2\pi }d\varphi C_{ij}(\varphi ,\theta ).$$

Using 〈c*φ*^2^〉=〈s*φ*^2^〉=1/2, 〈c*φ*^4^〉=〈s*φ*^4^〉=3/8 and 〈s*φ*^2^c*φ*^2^〉=1/8 (the odd power vanishes on average), we get, from equations (3.6) and (3.7), the following form of the Voigt matrix:

A 2 $$\left(\begin{array}{cccccc} C_{11}(\theta ) & C_{11}(\theta )-2C_{66}(\theta ) & C_{13}(\theta ) & 0 & 0 & 0\\ C_{11}(\theta )-2C_{66}(\theta ) & C_{11}(\theta ) & C_{13}(\theta ) & 0 & 0 & 0\\ C_{13}(\theta ) & C_{13}(\theta ) & C_{33}(\theta ) & 0 & 0 & 0\\ 0 & 0 & 0 & C_{44}(\theta ) & 0 & 0\\ 0 & 0 & 0 & 0 & C_{44}(\theta ) & 0\\ 0 & 0 & 0 & 0 & 0 & C_{66}(\theta ) \end{array}\right)$$

with the non-zero coefficients for this medium with transversely isotropic symmetry

A 3 $$\left.\begin{array}{rl} C_{11}(\theta ) & =\frac{1}{8}[A(3\text{s}\theta^{4}+8\text{c}\theta^{2})+3C\text{s}\theta^{4}+2(2L+F)\text{s}\theta^{2}(4-3\text{s}\theta^{2})],\\ C_{33}(\theta ) & =A\text{s}\theta^{4}+2(2L+F)\text{s}\theta^{2}\text{c}\theta^{2}+C\text{c}\theta^{4},\\ C_{44}(\theta ) & =\frac{1}{2}[(A+C-2F)\text{s}\theta^{2}\text{c}\theta^{2}+L(4\text{s}\theta^{4}-5\text{s}\theta^{2}+2)+N\text{s}\theta^{2}],\\ C_{66}(\theta ) & =\frac{1}{8}[(A+C-2F)\text{s}\theta^{4}+4L\text{s}\theta^{2}(2-\text{s}\theta^{2})+8N\text{c}\theta^{2}],\\ C_{13}(\theta ) & =\frac{1}{2}[A\text{s}\theta^{2}(2-\text{s}\theta^{2})+(C-4L)\text{s}\theta^{2}\text{c}\theta^{2}-2N\text{s}\theta^{2}+F(2\text{s}\theta^{4}-3\text{s}\theta^{2}+2)]. \end{array}\right\}$$

**(b) The average over *θ***

Next, we average the coefficients in equations (A 3) over *θ*. In agreement with equations (2.3) and (2.4), we use

A 4 $$\langle C_{ij}\rangle =\frac{1}{\left(1-\cos\theta_{0}\right)}\int_{0}^{\theta_{0}}d\theta \sin\theta \;C_{ij}(\theta ),$$

with *C*_*ij*_(*θ*) defined in equations (A 1) and (A 3). We use 〈c*θ*^2^〉=*X*/3, 〈s*θ*^2^c*θ*^2^〉=(2*X*−3*Y*)/15 and 〈c*θ*^4^〉=(*X*+*Y*)/5 with $X\equiv 1+\cos\theta_{0}+\cos\theta_{0}^{2}$ and $Y\equiv \cos^{3}\theta_{0}+\cos^{4}\theta_{0}^{2}$. We get

A 5 $$\left.\begin{array}{rl} \left\langle C_{11}\right\rangle & =\frac{1}{8\times 15}[A(45+19X+9Y)+3C(15-7X+3Y)+2(2L+F)(15+X-9Y)],\\ \left\langle C_{33}\right\rangle & =\frac{1}{15}[A(15-7X+3Y)+3C(X+Y)+2(2L+F)(2X-3Y)],\\ \left\langle C_{44}\right\rangle & =\frac{1}{30}[(A+C-2F)(2X-3Y)+3L(5-X+4Y)+5N(3-X)],\\ \left\langle C_{66}\right\rangle & =\frac{1}{8\times 15}[(A+C-2F)(15-7X+3Y)+4L(15-3X-3Y)+40NX],\\ \left\langle C_{13}\right\rangle & =\frac{1}{30}[A(15-3X-3Y)+(C-4L)(2X-3Y)+F(15+X+6Y)-10N(3-X)]. \end{array}\right\}$$

## Appendix B. Effective stiffness tensor for a partial girdle fabric

First, the elastic Voigt matrix is considered for *φ*=*π*/2,3*π*/2 (*δ*-functions in equation (2.9)) to make the configuration symmetric, so we consider

B 1 $$C_{ij}(\varphi ,\theta )\to C_{ij}(\theta )\equiv \frac{1}{2}\left[C\left(\frac{\pi }{2},\theta \right)+C\left(\frac{3\pi }{2},\theta \right)\right].$$

We get a Voigt matrix which has orthorhombic symmetry

B 2 $$\left(\begin{array}{cccccc} C_{11}(\theta ) & C_{12}(\theta ) & C_{13}(\theta ) & 0 & 0 & 0\\ C_{12}(\theta ) & C_{22}(\theta ) & C_{13}(\theta ) & 0 & 0 & 0\\ C_{13}(\theta ) & C_{13}(\theta ) & C_{33}(\theta ) & 0 & 0 & 0\\ 0 & 0 & 0 & C_{44}(\theta ) & 0 & 0\\ 0 & 0 & 0 & 0 & C_{55}(\theta ) & 0\\ 0 & 0 & 0 & 0 & 0 & C_{66}(\theta ) \end{array}\right)$$

with non-zero terms

B 3 $$\left.\begin{array}{rl} C_{11}(\theta ) & =A,\;C_{22}(\theta )=A\text{c}\theta^{4}+C\text{s}\theta^{4}+2(2L+F)\text{s}\theta^{2}\text{c}\theta^{2},\\ C_{33}(\theta ) & =A\text{s}\theta^{4}+C\text{c}\theta^{4}+2(2L+F)\text{s}\theta^{2}\text{c}\theta^{2},\\ C_{44}(\theta ) & =(A+C-2F)\text{s}\theta^{2}\text{c}\theta^{2}+L{\left(\text{c}\theta^{2}-\text{s}\theta^{2}\right)}^{2},\\ C_{55}(\theta ) & =L\text{c}\theta^{2}+N\text{s}\theta^{2},\;C_{66}(\theta )=L\text{s}\theta^{2}+N\text{c}\theta^{2},\\ C_{12}(\theta ) & =A\text{c}\theta^{2}-2N\text{c}\theta^{2}+F\text{s}\theta^{2},\;C_{13}(\theta )=A\text{s}\theta^{2}-2N\text{s}\theta^{2}+F\text{c}\theta^{2}. \end{array}\right\}$$

We now average over *θ*∈[0;*θ*_0_], according to equation (2.9) (the problem in *θ* is two dimensional), thus

B 4 $$\langle C_{ij}\rangle =\frac{1}{\theta_{0}}\int_{0}^{\theta_{0}}d\theta C_{ij}(\theta ).$$

We use 〈c*θ*^2^〉=(1−sinc2*θ*_0_)/2 and 〈c*θ*^4^〉=(3+4sinc2*θ*_0_+sinc4*θ*_0_)/8, with sincx≡sin⁡x/x (and sinc0=1). The resulting Voigt matrix is, with S_2_≡sinc2*θ*_0_, and S_4_≡sinc4*θ*_0_,

B 5 $$\left.\begin{array}{rl} \left\langle C_{11}\right\rangle & =A,\\ \left\langle C_{22}\right\rangle & =\frac{1}{8}[A(3+4{\text{S}}_{2}+{\text{S}}_{4})+C(3-4{\text{S}}_{2}+{\text{S}}_{4})+2(2L+F)(1-{\text{S}}_{4})],\\ \left\langle C_{33}\right\rangle & =\frac{1}{8}[A(3-4{\text{S}}_{2}+{\text{S}}_{4})+C(3+4{\text{S}}_{2}+{\text{S}}_{4})+2(2L+F)(1-{\text{S}}_{4})],\\ \left\langle C_{44}\right\rangle & =\frac{1}{8}[(A+C-2F)(1-{\text{S}}_{4})+4L(1+{\text{S}}_{4})],\\ \left\langle C_{55}\right\rangle & =\frac{1}{2}[L(1+{\text{S}}_{2})+N(1-{\text{S}}_{2})],\\ \left\langle C_{66}\right\rangle & =\frac{1}{2}[L(1-{\text{S}}_{2})+N(1+{\text{S}}_{2})],\\ \left\langle C_{12}\right\rangle & =\frac{1}{2}[(A-2N)(1+{\text{S}}_{2})+F(1-{\text{S}}_{2})],\\ \left\langle C_{13}\right\rangle & =\frac{1}{2}[(A-2N)(1-{\text{S}}_{2})+F(1+{\text{S}}_{2})],\\ \left\langle C_{23}\right\rangle & =\frac{1}{8}[(A+C-4L)(1-{\text{S}}_{4})+2F(3+{\text{S}}_{4})]. \end{array}\right\}$$

**(a) Comparison with [33]**

In their paper [33], Nanthikesan and Shyam Sunder considered a configuration equivalent to the partial girdle. For some reason, they took the *c*-axes in the horizontal plane with opening angle *ψ*_0_, and we take their results with ${\text{e}}_{1}^{N}={\text{e}}_{2}$, ${\text{e}}_{2}^{N}={\text{e}}_{3}$ and ${\text{e}}_{3}^{N}={\text{e}}_{1}$ (*N* refers to Nanthikesan and Shyam Sunder's notations). Their results on the mean elastic constants are denoted by $C_{ij}^{N}$ (eqn 15(b) in [33], not reported here for brevity) and are easily compared with our own. Owing to the correspondences between the coefficients (*ψ*_0_,*α*,*β*,*b*_1_,*b*_2_,*b*_3_) (defined after eqns (15b) in [33]) and our coefficients *S*_2_ and *S*_4_

B 6 $$\left.\begin{array}{rl} \frac{b_{1}}{2\psi_{0}} & =\frac{1}{8}(3+4S_{2}+S_{4}),\;\frac{b_{2}}{2\psi_{0}}=\frac{1}{8}(3-4S_{2}+S_{4}),\;\frac{b_{3}}{2\psi_{0}}=\frac{1}{8}(1-S_{4}),\\ \;\frac{\psi_{0}\pm \alpha }{2\psi_{0}} & =\frac{1}{2}(1\pm S_{2}),\;\frac{\psi_{0}/4\pm \beta }{2\psi_{0}}=\frac{1}{8}(1\pm S_{4})\;\mathrm{and}\;\frac{3\psi_{0}/2+2\beta }{2\psi_{0}}=\frac{1}{4}(3+S_{4}). \end{array}\right\}$$

It is now sufficient to rearrange the Voigt matrix according to the changes of axes $C_{ab}^{N}=\langle C_{ij}\rangle$ for *a*=1,2,3,4,5,6 (resp. *b*) corresponding to *i*=2,3,1,5,6,4 (resp. *a*). We get $C_{11}^{N}=\langle C_{22}\rangle$, $C_{22}^{N}=\langle C_{33}\rangle$, $C_{33}^{N}=\langle C_{11}\rangle$, $C_{44}^{N}=\langle C_{55}\rangle$, $C_{55}^{N}=\langle C_{66}\rangle$, $C_{66}^{N}=\langle C_{44}\rangle$, $C_{12}^{N}=\langle C_{23}\rangle$, $C_{13}^{N}=\langle C_{12}\rangle$ and $C_{23}^{N}=\langle C_{13}\rangle$, in agreement with eqns (15b) in [33] and with our equation (3.16).

## Appendix C. Effective stiffness tensor for a thick girdle fabric

We use the notations of figure 3, $\hat{\theta }$ and $\hat{\varphi }$ in $\left({\hat{\text{e}}}_{1},{\hat{\text{e}}}_{2},{\hat{\text{e}}}_{3}\right)$ for the ODF in equation (2.15). The derivation of ${\hat{C}}_{ab}(\hat{\theta })=1/(2\pi )\int_{0}^{2\pi }{\hat{C}}_{ab}(\hat{\theta },\hat{\varphi })$ is the same as in appendix A, resulting in equations (A 2) and (A 3)

C 1 $$\left.\begin{array}{rl} {\hat{C}}_{11}(\hat{\theta }) & ={\hat{C}}_{22}(\hat{\theta })=\frac{1}{8}[A(3\text{s}{\hat{\theta }}^{4}+8\text{c}{\hat{\theta }}^{2})+3C\text{s}{\hat{\theta }}^{4}+2(2L+F)\text{s}{\hat{\theta }}^{2}(4-3\text{s}{\hat{\theta }}^{2})],\\ {\hat{C}}_{33}(\hat{\theta }) & =A\text{s}{\hat{\theta }}^{4}+2(2L+F)\text{s}{\hat{\theta }}^{2}\text{c}{\hat{\theta }}^{2}+C\text{c}{\hat{\theta }}^{4},\\ {\hat{C}}_{44}(\hat{\theta }) & ={\hat{C}}_{55}(\hat{\theta })=\frac{1}{2}[(A+C-2F)\text{s}{\hat{\theta }}^{2}\text{c}{\hat{\theta }}^{2}+L(4\text{s}{\hat{\theta }}^{4}-5\text{s}{\hat{\theta }}^{2}+2)+N\text{s}{\hat{\theta }}^{2}],\\ {\hat{C}}_{66}(\hat{\theta }) & =\frac{1}{8}[(A+C-2F)\text{s}{\hat{\theta }}^{4}+4L\text{s}{\hat{\theta }}^{2}(2-\text{s}{\hat{\theta }}^{2})+8N\text{c}{\hat{\theta }}^{2}],\\ {\hat{C}}_{13}(\hat{\theta }) & =\frac{1}{2}[A\text{s}{\hat{\theta }}^{2}(2-\text{s}{\hat{\theta }}^{2})+(C-4L)\text{s}{\hat{\theta }}^{2}\text{c}{\hat{\theta }}^{2}-2N\text{s}{\hat{\theta }}^{2}+F(2\text{s}{\hat{\theta }}^{4}-3\text{s}{\hat{\theta }}^{2}+2)], \end{array}\right\}$$

with VTI symmetry in this frame (${\hat{C}}_{12}(\hat{\theta })={\hat{C}}_{11}(\hat{\theta })-2{\hat{C}}_{66}(\hat{\theta })$). The next average is now done on $\hat{\theta }\in [\pi /2-\xi_{0},\pi /2]$, according to equations (2.14) and (2.15), with

C 2 $$\langle {\hat{C}}_{ab}\rangle =\frac{1}{2\sin\xi_{0}}\int_{\pi /2-\xi_{0}}^{\pi /2+\xi_{0}}d\hat{\theta }\sin\hat{\theta }\;{\hat{C}}_{ab}(\hat{\theta }),$$

with *C*_*ij*_(*ξ*) given by equations (B 3). We use $\langle \text{c}{\hat{\theta }}^{2}\rangle =\sin^{2}\xi_{0}/3$, $\langle \text{c}{\hat{\theta }}^{4}\rangle =\sin^{4}\xi_{0}/5$ (thus, $\langle \text{s}{\hat{\theta }}^{2}\rangle =1-\sin^{2}\xi_{0}/3$ and $\langle \text{s}{\hat{\theta }}^{4}\rangle =1-2\sin^{2}\xi_{0}/3+\sin^{4}\xi_{0}/5$) leading to $\left\langle {\hat{C}}_{11}\rangle =\langle {\hat{C}}_{22}\right\rangle$, and

C 3 $$\left.\begin{array}{rl} \left\langle {\hat{C}}_{11}\right\rangle & =\frac{1}{8\times 15}[A(45+10{\text{s}\xi_{0}}^{2}+9{\text{s}\xi_{0}}^{4})+3C(15-10{\text{s}\xi_{0}}^{2}+3{\text{s}\xi_{0}}^{4})+2(2L+F)(15+10{\text{s}\xi_{0}}^{2}-9{\text{s}\xi_{0}}^{4})],\\ \left\langle {\hat{C}}_{33}\right\rangle & =\frac{1}{15}[A(15-10{\text{s}\xi_{0}}^{2}+3{\text{s}\xi_{0}}^{4})+3C{\text{s}\xi_{0}}^{4}+2(2L+F)(5{\text{s}\xi_{0}}^{2}-3{\text{s}\xi_{0}}^{4})],\\ \left\langle {\hat{C}}_{44}\right\rangle & =\langle {\hat{C}}_{55}\rangle =\frac{1}{30}[(A+C-2F)(5{\text{s}\xi_{0}}^{2}-3{\text{s}\xi_{0}}^{4})+3L(5-5{\text{s}\xi_{0}}^{2}+4{\text{s}\xi_{0}}^{4})+5N(3-{\text{s}\xi_{0}}^{2})],\\ \left\langle {\hat{C}}_{66}\right\rangle & =\frac{1}{8\times 15}[(A+C-2F)(15-10{\text{s}\xi_{0}}^{2}+3{\text{s}\xi_{0}}^{4})+12L(5-{\text{s}\xi_{0}}^{4})+40N{\text{s}\xi_{0}}^{2}],\\ \left\langle {\hat{C}}_{13}\right\rangle & =\frac{1}{30}[3A(5-{\text{s}\xi_{0}}^{4})+(C-4L)(5{\text{s}\xi_{0}}^{2}-3{\text{s}\xi_{0}}^{4})-10N(3-{\text{s}\xi_{0}}^{2})+F(15-5{\text{s}\xi_{0}}^{2}+6{\text{s}\xi_{0}}^{4})], \end{array}\right\}$$

and $\left\langle {\hat{C}}_{12}\rangle =\langle {\hat{C}}_{11}\rangle -2\langle {\hat{C}}_{66}\right\rangle$ with $\text{s}\xi_{0}\equiv \sin\xi_{0}$. It is now sufficient to use the correspondences resulting from equation (2.12) to HTI symmetry with respect to **e**_1_ (see equations (3.21) and (3.22)),

C 4 $$\left.\begin{array}{rl} \left\langle C_{11}\right\rangle & =\langle {\hat{C}}_{33}\rangle ,\;\langle C_{22}\rangle =\langle C_{11}\rangle =\langle {\hat{C}}_{11}\rangle ,\;\langle C_{44}\rangle =\langle {\hat{C}}_{66}\rangle ,\\ \left\langle C_{55}\right\rangle & =\langle C_{66}\rangle =\langle {\hat{C}}_{44}\rangle \;\mathrm{and}\;\langle C_{12}\rangle =\langle {\hat{C}}_{13}\rangle . \end{array}\right\}$$
